# Likely Role of APOBEC3G-Mediated G-to-A Mutations in HIV-1 Evolution and Drug Resistance

**DOI:** 10.1371/journal.ppat.1000367

**Published:** 2009-04-03

**Authors:** Patric Jern, Rebecca A. Russell, Vinay K. Pathak, John M. Coffin

**Affiliations:** 1 Department of Molecular Biology and Microbiology, Tufts University School of Medicine, Boston, Massachusetts, United States of America; 2 HIV Drug Resistance Program, National Cancer Institute-Frederick, Frederick, Maryland, United States of America; University of Geneva, Switzerland

## Abstract

The role of APOBEC3 (A3) protein family members in inhibiting retrovirus infection and mobile element retrotransposition is well established. However, the evolutionary effects these restriction factors may have had on active retroviruses such as HIV-1 are less well understood. An HIV-1 variant that has been highly G-to-A mutated is unlikely to be transmitted due to accumulation of deleterious mutations. However, G-to-A mutated hA3G target sequences within which the mutations are the least deleterious are more likely to survive selection pressure. Thus, among hA3G targets in HIV-1, the ratio of nonsynonymous to synonymous changes will increase with virus generations, leaving a footprint of past activity. To study such footprints in HIV-1 evolution, we developed an *in silico* model based on calculated hA3G target probabilities derived from G-to-A mutation sequence contexts in the literature. We simulated G-to-A changes iteratively in independent sequential HIV-1 infections until a stop codon was introduced into any gene. In addition to our simulation results, we observed higher ratios of nonsynonymous to synonymous mutation at hA3G targets in extant HIV-1 genomes than in their putative ancestral genomes, compared to random controls, implying that moderate levels of A3G-mediated G-to-A mutation have been a factor in HIV-1 evolution. Results from *in vitro* passaging experiments of HIV-1 modified to be highly susceptible to hA3G mutagenesis verified our simulation accuracy. We also used our simulation to examine the possible role of A3G-induced mutations in the origin of drug resistance. We found that hA3G activity could have been responsible for only a small increase in mutations at known drug resistance sites and propose that concerns for increased resistance to other antiviral drugs should not prevent Vif from being considered a suitable target for development of new drugs.

## Author Summary

The search for new drugs to battle HIV-1 infections is a continuing struggle. APOBEC3G proteins have been shown to deaminate C-residues in HIV-1 minus strand DNA during its synthesis, resulting in G-to-A mutations in the RNA genome. The HIV-1 Vif protein has evolved to counteract APOBEC3G and thereby escape these frequently deleterious mutations, making Vif an attractive target for new drugs. However, a partial block of Vif could result in an increased although low-level HIV-1 G-to-A mutation rate. Here we investigated APOBEC3G mutation footprints in HIV-1 evolution and the potential risk for known drug resistance from sublethal G-to-A mutations. Using computer simulations, the accuracies of which were verified by infection experiments, we detected evolutionary APOBEC3G mutation footprints in the HIV-1 genome. We predict that the risk that APOBEC3G-induced G-to-A mutations will cause drug resistance is very low. We therefore propose that concerns for increased resistance to other antiviral drugs should not prevent Vif from being considered a suitable target for development of new drugs.

## Introduction

The human and mouse APOBEC3 (A3) protein family members, hA3F and G and mA3, respectively, are well-studied host factors that restrict retrovirus replication. [1]–[3]. These components of the innate cellular defense system inhibit retroviral propagation by inducing deamination of C-to-U residues in the negative strand of retroviral DNA during reverse transcription [4], resulting in a mutated provirus with biased G-to-A changes on the plus strand [5]–[8]. Due to the specificity of A3 for single-stranded DNA, the frequency of induced mutations forms an increasing gradient in the genome from the primer binding site (PBS) to the polypurine tract (PPT) [9]. In the case of HIV-1, whose genome contains a central PPT (cPPT), this effect results in dual gradients of G-to-A mutations from the PBS to the cPPT and the cPPT to the PPT [10]. A role of A3-mediated defense in more distant retroviral evolution became apparent when we recently showed that mA3 likely contributed to inactivation of the infectivity of some types of endogenous MLV at the time of their integration into the host germline around a million years ago [11]. Taken together, these observation show that A3 was active as an antiviral factor in the distant evolutionary past, and that it is so today. However, the effects these APOBEC3 restriction factors have had on the evolution of actively replicating retroviruses such as HIV-1 are less clear.

The present study was undertaken to look for a signal of past A3-induced G-to-A changes in the sequence of modern day HIV-1 genomes. We hypothesized that the observed variation in G∶A ratios in different viruses and in their sensitivities to A3 [5],[12],[13] is partly an effect of earlier A3-induced mutation and selection pressure on the virus genomes. Highly G-to-A mutated sequences are unlikely to be transmitted due to deleterious mutations. However, less efficient A3 activity, and thus fewer G-to-A mutations, could allow virus transmission, although with reduced efficiency. In this scenario, mutated A3 target sequences within which G-to-A mutations are the least deleterious are more likely to survive purifying selection pressure. This model predicts that as mutations accumulate with time, the ratio of nonsynonymous (NS) to synonymous (S) sites in probable A3 target sequences will increase with increasing virus generations more rapidly than at sites that are not A3 targets.

To study the potential of human APOBEC3G (hA3G) to affect HIV-1 evolution, we developed an *in silico* model that takes into account the observed mutation gradient and the probabilities of hA3G-mediated mutation at each site, derived from the sequence contexts of mutation sites (hereafter referred to as “contexts”) found in the literature [9],[14]. After adjustment for location in the provirus in our simulations, we found that the frequency of G-to-A mutations introduced into HIV-1 could be partly explained by the virus's genetic profile as well as its distribution of probable target sequence contexts. Our simulation approach is supported by independent experimental results obtained by passaging HIV-1 under conditions that permit a high level of A3G-induced mutation. Further analyses showed that the ratio of NS to S-mutations at hA3G target sites has increased during HIV-1 evolution compared to random controls, implying an evolution where A3 sites have had higher mutation rates than non-A3 sites and therefore been subjected to greater influence from purifying selection. We also analyzed the potential of low-level hA3G-mediated G-to-A mutations to give rise to naturally occurring drug resistance mutations.

## Materials and Methods

### Human APOBEC3G mutation site contexts and probabilities

To calculate the likelihood of hA3G-mediated G-to-A mutation at different sites in HIV-1, we analyzed mutation site contexts surrounding 1324 observed G-to-A mutations attributed to hA3G from the literature [9],[14]. Given the variability in these data and to minimize false predictions, we used our previous experience with mutation site context analyses [11] and included as many significant sites as possible (Figure S1). Thus, we collected sequences up to 3 bases 5′ and 5 bases 3′ of the G-to-A mutations. Using nucleotide frequencies at each offset position from the mutation target G-nucleotide, we constructed a position-specific scoring matrix (PSSM, Figure S1) using odds ratios (i.e., observed relative to expected nucleotide frequencies) and similar to the one derived for our previous study on endogenous nonecotropic MLVs [11]. Due to limited representation of observed non-A3G mediated G-to-A mutation sites in the literature, we included an artificial nucleotide frequency background to compensate for sampling artifacts using a previously described pseudo count method (Figure S1) [15]. Using automated PERL scripts we simulated a collection of 10^6^ random mutation site contexts based on nucleotide frequencies observed in HIV-1. These data were used to derive a score distribution and a cumulative frequency distribution for probability calculation of context scores. Scores for the previously described G-to-A contexts were calculated and compared to the newly calculated score and cumulative frequency distributions. An analysis of G-to-A mutation site contexts collected from endogenous MLVs [11] supported this putative cutoff for likely A3 targets (data not shown). In addition to our simulations using all hA3G targets, we also included separate analyses using the 80% probability cutoff for introduction of G-to-A mutations (see below).

### Simulated retrovirus evolution from G-to-A mutations

**Figure 1:**
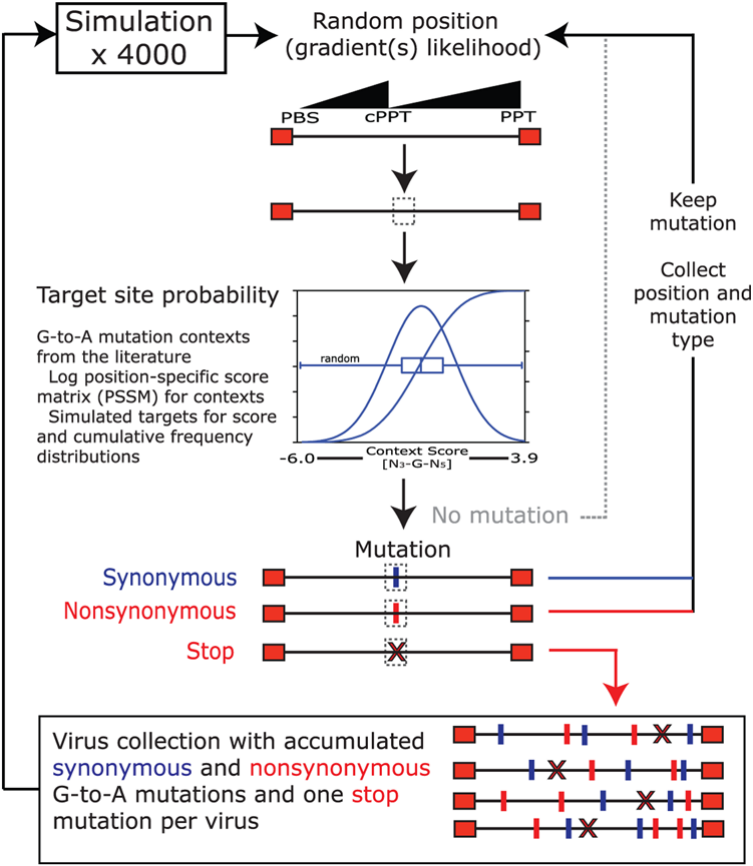
Simulation strategies and analyses. A mutation position was randomly selected along the provirus, considering dual likelihood gradients, and analyzed for its surrounding context score and fitness to a hA3G target using simulated score and cumulative frequency distributions (as shown in the graph; see Figure 2A). A random number between 0 and 1 was chosen. If the context likelihood at the chosen site exceeded that value, a G-to-A mutation was introduced at that site. Failure to mutate initiated another attempt for a random mutation position. For a successful mutation, its position and synonymous or nonsynonymous change in amino acid were recorded and the mutated sequence was subjected to another round of mutation. This process was iterated until a stop codon was recorded in any ORF, at which point the sequence was collected into a database and a new simulation was initiated. Four thousand independent simulations were performed for each run.

Mutations were simulated in HIV-1 (AF033819) using automated PERL scripts as outlined in Figure 1. Briefly, a mutation position was randomly selected using the previously observed dual gradients of increasing likelihood for mutation in the PBS-cPPT and cPPT-3′PPT regions [9],[10]. A score for the sequence context surrounding each randomly selected G-nucleotide was compared against cumulative frequency distributions of simulated random viral hA3G target contexts, and used to test whether a G-to-A mutation should be introduced at that site. If the context likelihood exceeded a random number between 0 and 1 a G-to-A mutation was introduced at that site. Failure to introduce a mutation initiated a new attempt at another random position. Information from successful mutations, including their location and whether they were synonymous (S) or nonsynonymous (NS) (i.e. unchanged and changed amino acid respectively) – was collected and followed by another round of simulated G-to-A mutation. Simulations were repeated until a stop codon was introduced into any gene or a normal ATG initiation codon was altered, after which the HIV-1 sequence with accumulated G-to-A mutations was collected into a database. In this way, we collected 4000 independent genomes with accumulated G-to-A mutations. All simulation rounds were performed using either contexts with probabilities over 80% or all G-nucleotides independent of context.

### Test of evolutionary G-to-A mutation footprints in HIV-1

To analyze the potential evolutionary footprints of hA3G-mediated G-to-A mutations in HIV-1 we used automated PERL scripts to test the ratios between the summarized probabilities of nonsynonymous to synonymous G-nucleotide contexts. To look for mutations that might have occurred during the evolutionary history of the virus, the same analysis was performed for putative ancestral G-nucleotide targets observed as A-nucleotides in the virus. Ratios were compared using a χ^2^ test (1 degree of freedom) for HIV-1 (AF033819) and HIVcon (group M consensus sequence derived from subgroup gene alignment consensus sequences) downloaded from www.hiv.lanl.gov.

### Potential HIV-1 drug resistance from hA3G-mediated G-to-A mutations

Locations of known HIV-1 protease inhibitor (PI), nucleoside RT inhibitors (NRTI), non-nucleoside RT inhibitor (NNRTI), integrase inhibitor (INI), and fusion inhibitor (FI) resistance mutation sites were collected from the literature [16] and the HIV drug resistance database (http://hivdb.stanford.edu). We then analyzed all G-nucleotide contexts and used their calculated probabilities in our *in silico* model to estimate the potential likelihood for hA3G-mediated G-to-A mutations leading to drug resistance.

### Experimental assessment of hA3G-induced mutations in HIV-1

Highly mutated HIV-1 samples were obtained from a passaging experiment. A detailed description of these studies [17] is available upon request. In brief, the YRHHY>A5 mutation at amino acids 40 to 44, which renders HIV-1 Vif unable to efficiently bind to hA3G but still be effective against hA3F [18], was inserted into the replication-competent HIV-1 plasmid pNL4-3 (AF324493) using overlapping PCR to generate pNL4-3-YRHHY>A5. For virus production, 4×10^6^ 293T cells were transfected with 20 µg of either pNL4-3 or pNL4-3-YRHHY>A5 and 1.2 µg pGL, which expresses the green fluorescent protein from a cytomegalovirus immediate early promoter (Invitrogen) to determine transfection efficiency. The virus-containing supernatant was harvested 48-hours after transfection, and assayed for RT activity. On day one of infection, 1×10^6^ CEM cells were infected with 200 µl of virus supernatant (1000 RT units of virus) for five hours. The volume of medium was then increased to 5 ml. A 4 ml aliquot of cells and virus-containing supernatant was removed at two day intervals post infection (days 3, 5, 7 etc.), and stored at −70°C for subsequent analysis. A 4 ml aliquot of fresh CEM-CM (complete medium, RPMI+10% fetal calf serum+1% penicillin-streptomycin, in which the CEM cells were maintained). was then added to the remaining 2 ml cell and virus suspension and the sample incubated for another 2 days.

PCR reactions using 2 µl of extracted DNA from the infected cells, 1 µl High Fidelity Platinum Taq polymerase (Invitrogen) and 20 pmoles each of Vif F (5′CAGGGAGATTCTAAAAG3′) and Vif R (5′GGATAAACAGCAGTTGTTGC3′) primers were performed at 98 C for 2 minutes, followed by 30 cycles at 98 C for 30 seconds, 55 C for 30 seconds and 72 C for one minute. Final PCR product extension was performed at 72 C for 10 minutes. The PCR product was then cloned into the TOPO TA vector (Invitrogen) and the individual clones were sequenced using the primer NL43 seq 4921F (5′GAGATCCAGTTTGGAAAGGAC3′).

### Evaluation of the *in silico* model

Mutation site contexts from the *in vitro* experiments described above were collected using automated PERL scripts as described earlier [11]. Scores and likelihoods were calculated in our current model. In these experiments, G-to-A mutations were considered either natural RT errors or hA3G-mediated changes since the YRHHY>A5 *vif* mutant is still capable of inhibiting hA3F [18]. To evaluate our *in silico* model, we simulated the number of mutations we observed *in vitro* on the same *vif* region of pNL4-3 (AF324493; positions 5041–5770). We then isolated all contexts surrounding simulated G-to-A mutations using our automated PERL scripts and calculated scores and likelihoods for direct comparison to the *in vitro* results.

## Results

### Simulated HIV-1 evolution due to G-to-A mutations

**Figure 2:**
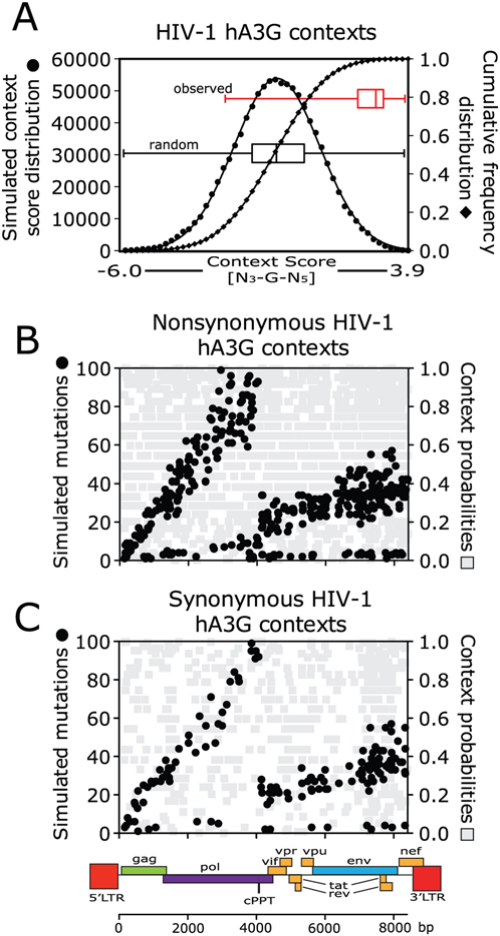
Human APOBEC3G target context probabilities and simulation results in HIV-1. (A) The distribution of scores for 10^6^ random sequence contexts derived from observed nucleotide frequencies in HIV-1 extending 3 bases upstream and 5 bases downstream of the target base is shown by the black circles. The black diamonds show the cumulative frequency distribution, and a boxplot representing this distribution (median, flanked by boxes indicating first and third quartiles and whiskers for high and low values) is also shown in black. The distribution of corresponding context scores for previously observed sites of hA3G-mediated G-to-A mutations in HIV-1 [9],[14] is shown by the red boxplot. (B, C) Frequency of accumulated nonsynonymous (B) and synonymous mutations (C) at contexts with >80% probability derived from 4000 independent simulations of virus evolution (see Figure 1) are shown (in black) relative to the HIV-1 provirus. The simulated mutations show the expected dual mutation gradients [10], PBS-cPPT, and cPPT-3′PPT. The differences in slope between 5′- and 3′-gradients reflect the different gradient lengths along the virus sequence. Grey symbols show the distribution of all potential G-to-A mutation sites as a function of their relative probabilities derived from PSSM scores, thus verifying that the distributions observed are not due to a nonuniform distribution of high probability hA3G targets.

Using automated PERL scripts and previously identified hA3G-induced mutations [9],[14], we extracted sequence contexts extending 3 bases upstream and 5 bases downstream of each potential G-to-A mutation position in the HIV-1 genome and constructed a Log position-specific scoring matrix (PSSM, Figure S1). We next created a random set of 10^6^ mutation site contexts (Figure 2A, circles) based on the HIV-1 base composition and plotted the histogram and cumulative frequencies of that distribution (Figure 2A, diamonds). This analysis yielded an approximately normal distribution with a mean score of −0.7 and a standard deviation of 1.5 (black boxplot). To determine the pattern of actual mutations, we then tested context scores of previously observed G-to-A mutation sites [14] against this distribution. The distribution of calculated probabilities of these mutation “hotspots” [14] was significantly skewed relative to random, with a mean of 2.3 and a standard deviation of 1.1 (Figure 2A; red boxplot), with 96% of mutations at sites with a score of 80% or greater, suggesting that a score threshold of 80% would capture the large majority of actual hA3G-mutation targets. We also found support for an 80% cutoff when G-to-A mutation site contexts collected from endogenous MLVs [11] were analyzed in the same way using simulated score distributions.

To estimate the potential of hA3G-mediated mutations to contribute to HIV-1 evolution, we used our *in silico* model (Figure 1) to simulate stochastic accumulation of G-to-A mutations in the genome before an obviously lethal mutation (introduction of a stop codon or loss of an initiation codon) appeared in any gene (Figure 2B and C). The simulation took into account both the dual hA3G mutation gradients (PBS-cPPT and cPPT-3′PPT) and the target probability at each G-nucleotide calculated from the surrounding sequence context. A collection of 4000 HIV-1 sequences – with nonsynonymous (NS, Figure 2B) and synonymous (S, Figure 2C) mutations accumulated until one lethal mutation was introduced – was analyzed. Most of the simulated mutations displayed the expected dual gradient (black circles). Very infrequent mutations at the bottom of the graphs result from contexts that are at or very close to the probability cutoff. To ensure that this pattern did not reflect a nonuniform distribution of high probability hA3G targets, we also plotted the distribution of context probability scores for all G-residues. As can be seen (Figure 2B and C, gray squares), there is no indication of an uneven distribution of context probabilities across the HIV-1 provirus.

**Figure 3:**
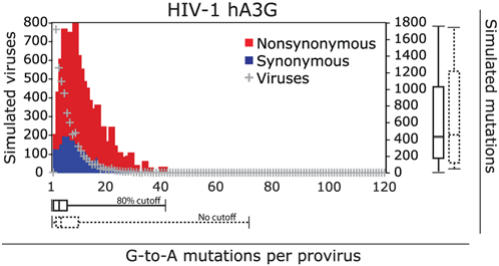
Simulation of hA3G-mediated HIV-1 evolution. A total of 4000 *in silico* G-to-A mutated HIV-1 sequences is indicated by grey crosses plotted as the number of genomes vs. number of mutations per genome. Bars show nonsynonymous (red) and synonymous (blue) mutations. Boxplots below show the distributions of viruses with hA3G-induced mutations, with the median value flanked by boxes indicating the quartiles and whiskers indicating the highest and lowest number of mutations in the distributions. Solid boxplots show distributions from simulations using 80% probability cutoffs for mutations and dashed boxplots show equivalent results considering all context probabilities. Complementing boxplots on the right show distributions of accumulated mutations from the simulations using hA3G.

The distribution of the number of accumulated G-to-A changes per provirus resulting from our simulations is presented in Figure 3. For comparison, simulations were also repeated in which all G-nucleotide sites were considered for successful mutations and compared to those derived using the 80% probability cutoff (Figure 2 and above). As expected from our model, without selection pressure on the virus sequence, NS (red) to S (blue) mutation ratios were consistent (approximately 3∶1) independent of the number of mutations that were introduced into the viruses. Further, the numbers of random G-to-A mutations introduced before a stop codon appeared averaged about 1.6 S and 4.0 NS mutations, but some individual genomes accumulated as many as 40 substitutions when an 80% hA3G target probability cutoff was used (Figure 3). This value increased to 72 when all G-nucleotides were considered. Thus, when all sites were used in simulations, mutations were more widespread and high scoring hA3G target contexts accumulated slightly fewer mutations than in simulations where a probability cutoff was used (Figure S2). This pattern reflects the fact that higher scoring sites have a larger proportion of potential stop codons resulting from G-to-A mutations. Together, these simulation results suggest that a moderate level of G-to-A mutations is tolerable in the virus sequence during its evolution without causing significant harm (i.e. premature stops), although the significance of other NS mutations was not assessed in our simplified *in silico* model.

### G-to-A mutation footprints in retrovirus evolution

Given the number of G-to-A mutations that could be introduced in our *in silico* model, it is conceivable that a low-level of hA3G-mediated mutations has been introduced into the HIV-1 sequence during its recent history and that these mutations might still be distinguishable from the more random RT-induced G-to-A mutations.

**Figure 4:**
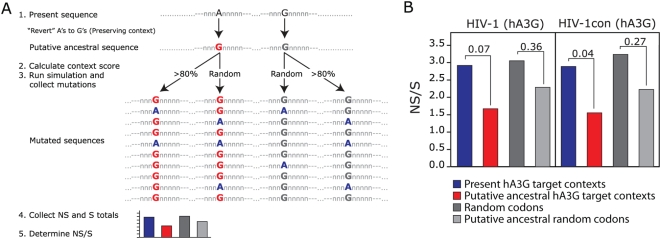
G-to-A mutation footprints formed during the HIV-1 evolutionary past. (A) The principle of this analysis. Briefly, scores were summarized for contexts surrounding G-nucleotides and A-nucleotides (treated as putative ancestral G) and analyzed for nonsynonymous (NS) or synonymous (S) change if mutated G-to-A. For comparison, NS/S ratios were also calculated for random codons derived from nucleotide frequencies at codon positions found in the provirus ORFs. (B) The NS/S ratio differences in a modern HIV-1 subtype B sequence (AF033819) are shown in the left panel and HIVcon (group M consensus sequence derived from the individual subgroup consensus sequences) are in the right panel. The NS/S ratio of hA3G targets (blue) are compared to corresponding putative ancestral targets (red) with an A-nucleotide at the target site as well as to expected ratios derived from G- and A-nucleotide frequencies and positions in randomly selected codons. P-values from the χ^2^ distribution (1 degree of freedom) are shown above the bars.

To investigate this possibility, we used our *in silico* model (Figure 2A) to calculate the likelihood of each G-nucleotide context fit to be a hA3G target as outlined in Figure 4A. Briefly, we summarized the likelihood scores for each G-nucleotide context and tested for mutation type when changed into an A-nucleotide. We then calculated a ratio of NS to S sites (Figure 4A). In the same way, we summarized likelihood scores for currently residing A-nucleotides, which we treated as putative ancestral G-nucleotides. Evolutionary theory predicts that the least costly mutations will have a higher chance to become fixed in the viral sequence and that deleterious mutations, i.e. mostly NS, will naturally be sorted out from the virus population due to purifying selection pressure. Conversely, among target sequences in which the target G-nucleotide has been changed to an A (referred to here as “putative” targets), the NS/S ratio should increase with time. Thus, the action of A3 on ancestral virus should have left a footprint on the viral genome in the form of an increased NS/S ratio at A3G targets relative to putative ancestral targets (Figure 4A). In search of such a footprint, we tested a modern HIV-1, subtype B sequence for the score and likelihood ratios at potential hA3G target sites and found an increased NS/S ratio (p = 0.07, χ^2^ test) when compared to the cognate ratios at putative ancestral sites (Figure 4B). Indeed, analyses using the group M consensus sequence reconstructed from subgroup gene consensus sequences (HIVcon), which most likely has eliminated some background variation, supported our finding with a higher level of significance (p = 0.04). Increasing the target score cutoff led to slightly higher ratios for HIV-1, whereas a decreased cutoff had the opposite effect on both sequences (data not shown). To ensure that differences in NS/S ratios between currently observed G-nucleotides and putative ancestral hA3G mutation site contexts were not artifacts from random HIV-1 RT errors, we repeated our analyses by random sampling of codons from the virus sequences while not considering context scores or likelihoods. In these controls we found no significant differences in nonsynonymous to synonymous ratios between the current and putative ancestral G-nucleotides (Figure 4B). Thus, we propose that hA3G-induced mutation has had a significant influence on the HIV-1 genome in its evolutionary past.

### Potential HIV-1 drug resistance from hA3G-mediated G-to-A mutations

Our analysis implies that, over its evolutionary history, HIV-1 has been subjected to sublethal G-to-A mutations, which have not had sufficient effect to stop virus proliferation and transmission. An imperfectly functioning Vif protein might explain this result. Vif has become an interesting target for new anti-viral drugs [19]–[21] since inhibition of its activity could increase the chance for hA3G to more efficiently restrict virus spread. However, suboptimal blocking of Vif might also lead to an increase in sublethal hA3G-induced G-to-A mutations, and potentially more rapid appearance of drug resistance. To test this possibility, we analyzed the G-nucleotide contexts in the major HIV-1 genes *gag*, *pro*, *pol* and *env* (Figure S2). We hypothesized that combinations of HIV-1 inhibitory drugs, including a potential Vif-targeting drug, could lead to an increased selection pressure for HIV-1, which might then benefit from low-level hA3G-mediated G-to-A mutations, allowing it to escape other drugs like protease-, RT-, integrase- and fusion-inhibitors.

**Figure 5:**
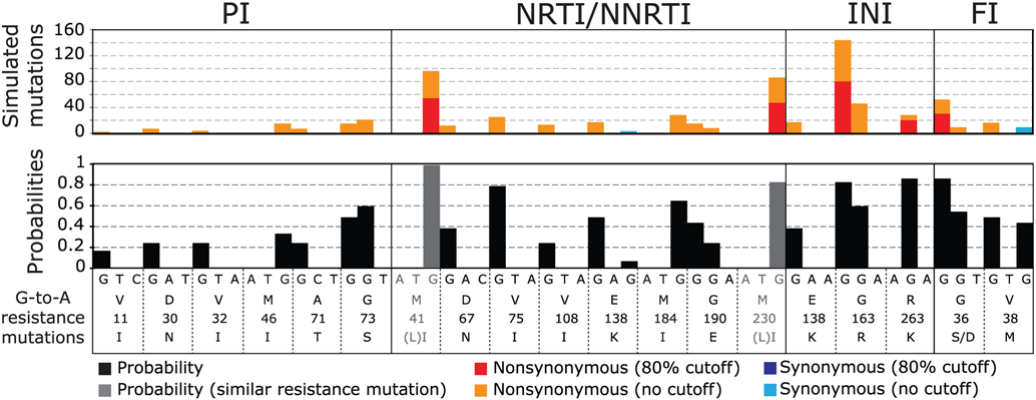
Drug resistance mutations potentially caused by G-to-A mutations. Codons for known mutation sites are concatenated from full mutation site contexts in the HIV-1 *gag*, *pol* and *env* sequences (Figure S2). Lower panel: black bars indicate calculated probabilities at G-to-A mutation positions (derived from Figure 2A). The upper panel shows stacked bars for accumulated mutations at each position from simulations using the 80% probability cutoff (nonsynonymous red or synonymous blue) or all probabilities (orange or light blue). Abbreviations: PI, protease inhibitors; NRTI, nucleoside RT inhibitors; NNRTI, non-nucleoside RT inhibitors; INI, integrase inhibitors; FI, fusion inhibitors.

To test this possibility, we screened a total of about 52,000 mutations resulting from our *in silico* simulations using either 80% probability cutoffs or all G-nucleotide context probabilities (Figure 3), our analyses showed a total of 695 mutations introduced at known drug resistance sites (Figure 5 and Figure S2). However, in no case was the frequency of mutations at sites with high probability scores greatly different from the probability for all G-to-A mutations regardless of score. Thus, it seems unlikely that increased drug resistance would arise as a significant side effect of an increased, but still low-level, hA3G-induced mutation rate resulting from using Vif as an anti HIV-1 drug target. However, caution is indicated where, due to an altered selection pressure from anti-viral regimens used in combination, multiple G-to-A mutations in adjoining contexts could potentially lead to new drug resistance (Figure S2).

### Experimental evaluation of the model

Our current *in silico* model is based on previously published experimental data [9],[10],[14]. To test the role of hA3G more directly, we performed *in vitro* passaging experiments on CEM cells using a previously described HIV-1 *vif* mutant [18] that was previously shown to have lost the ability to inhibit hA3G, but could still inhibit hA3F; therefore G-to-A mutations were considered to result from either RT-induced errors or hA3G-mediated changes [17],[18]. Western blotting analysis indicated that the T cell line used did not express detectable levels of hA3F (data not shown), further supporting the view that hA3F did not significantly contribute to the pool of G-to-A mutations analyzed. We also analyzed another *vif* mutant which is unable to block the activity of hA3F but can block the activity of hA3G (DRMR>A4). This mutant replicated with kinetics that were indistinguishable from those of the wild type virus (unpublished observations). Thus, we are confident that these cells express little or no hA3F. To assess the distribution of accumulated mutations, we passaged the virus sequentially for three rounds over three months, and then sequenced the *vif* region (corresponding to positions 5041–5770 of pNL4-3), amounting to about 166,000 nucleotides from 227 separate genomes. We identified a total of 585 G-to-A mutations and calculated scores and probabilities for their respective hA3G mutation site contexts. In the raw sequence data [17],[18], we noted that approximately 87% of the mutations were in GG dinucleotide contexts, agreeing with frequencies observed by us and others for vif-defective viruses produced from cells that exclusively express hA3G (transfected 293T cells).

**Figure 6:**
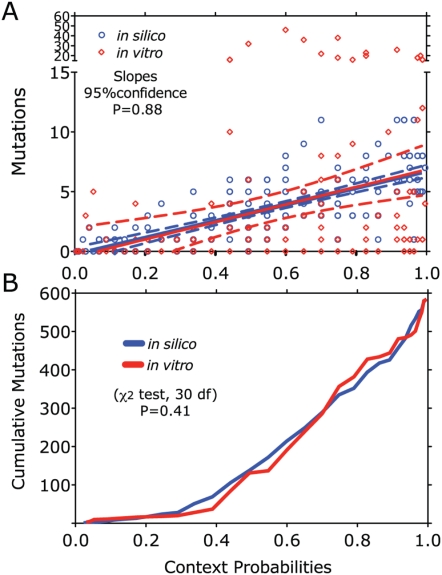
Validation of the *in silico* model for hA3G-mediated G-to-A mutations in HIV-1. (A) The number of mutations in HIV-1 (pNL4-3 *vif*-region) from *in vitro* experiments plotted against their respective calculated probability scores in red symbols. The trend is presented as a solid red line flanked by dashed lines indicating the 95% confidence interval. An equal number of mutations was simulated over the same sequence using our *in silico* model (see Figure 1 and Figure 2) is shown by the blue symbols, blue trend line and 95% confidence interval. (B) The cumulative distributions of contexts mutated *in silico* (blue) and *in vitro* (red) plotted against their calculated probabilities.

To evaluate our *in silico* model predictions we simulated the same number of mutations observed *in vitro* into the same pNL4-3 sequence. A comparison of the observed and predicted frequencies of G-to-A mutations as a function of context probability is shown in Figure 6A, and the corresponding cumulative distributions are shown in Figure 6B. We could not detect any significant differences in the mean values (P = 0.41, χ^2^ test, 30 degrees of freedom) between our *in silico* predictions and observations from *in vitro* experiments. The larger variation observed *in vitro* (red confidence intervals and red outliers) compared to the *in silico* results (blue confidence intervals) indicates that other factors such as nucleic acid structure might also influence the efficiency of hA3G-mediated mutation. However, given the similarity of the cumulative distribution, this contribution is likely to be small compared to the primary sequence likelihood used in our predictions, implying that our model is capable of accurately capturing hA3G-mediated mutagenesis during HIV replication.

## Discussion

Human A3F and A3G are potent antiviral factors whose activities, if not blocked by Vif, are capable of causing high levels of G-to-A mutations in newly made viral DNA. However, their role in the genesis of naturally occurring mutations is unclear. In this report, we have built an *in silico* model to simulate G-to-A mutations and to analyze their potential role in HIV-1 evolution and drug resistance. We have found that hA3G activity acting on prior generations of virus has left detectable footprints in the HIV-1 genome. Although there are at least ten human APOBEC members (reviewed in [2],[12],[22]), including at least one more potential HIV-1 restriction factor, hA3H [23],[24]; we have chosen to focus on the effects of hA3G on HIV-1 to limit our predictions and simplify our model in order to reach realistic conclusions. We also elected to include a wider mutation site context than the more commonly used GG-dinucleotide, consistent with our previous studies of nonrandom mA3 targets in endogenous nonecotropic MLV [11], to distinguish hA3G-induced G-to-A changes from randomly introduced HIV-1 RT errors.

G-to-A hypermutation in HIV-1 is clearly capable of limiting virus proliferation due to the introduction of both missense and nonsense mutations, which can be directly lethal to the virus [7],[8]. The origin of G-to-A changes and their contribution to the bias and A-richness of the HIV-1 genome have been studied, as has the role of hA3G as a potential driver of HIV-1 evolution, although mutations introduced by RT are likely to be of greater general importance in the evolution of drug-resistant variants [25]. The hypothesis that hA3G has played an important role in HIV-1 evolution was also discussed in another recent study of HIV-1 nucleotide and codon usage bias [26].

To address the potential role of hA3G-mediated G-to-A mutations, as distinguished from random HIV-1 RT-introduced errors, in HIV-1 evolution, we analyzed individual mutation sites in hA3G target contexts, calculated from independent experimental data. To minimize bias due to purifying selection, the impaired vif gene was chosen for analysis of G-to-A mutation frequency. Another practical reason was that we needed to verify with each sequence that the Vif inactivating mutation was not reverted to regain Vif function. Thus, analyzing sequences from the Vif/Vpr region allowed us to verify the Vif-deficient phenotype. When the results obtained from this *in silico* analysis were compared with the accumulation of G-to-A mutations in *vif(-)* HIV-1, passaged repeatedly in CEM cells, we saw no significant difference in mean number of mutations as a function of their context score, although the variance of this value was much greater in the latter analysis, perhaps reflecting effects of secondary structure or some other feature on the A3G mediated mutation frequency. It should be emphasized that, although we used the same total number of mutations observed *in vitro* for our simulation, the *in silico* model was otherwise completely independent of the *in vitro* study, yet the two did not give significantly different results. Thus, the similarity between experiment and simulation supports the accuracy of our model, as well as its suitability for estimating mutational events. Further support for our hA3G footprint observations in HIV-1 and our estimates of moderately high tolerance of G-to-A changes before introduction of deleterious stop mutations in our simulations could also be observed in a recent study of genetic variation in early founder HIV-1 virus populations from primary infections [27].

Our results imply that although hA3G-mediated G-to-A mutations likely have had a detectable effect on HIV-1 evolution that is traceable through mutational footprints Indeed, several studies of endogenous retroviruses from different genera and host species have recently shown evolutionary effects caused by A3-introduced G-to-A mutations, some of which likely contributed to provirus inactivation [11],[28],[29]. However, it is likely that other mutagens, including other A3 proteins, as well as HIV-1 RT are also important contributors to the nucleotide bias in the virus genome [25],[26].

The HIV-1 Vif protein has drawn interest as a drug target since interfering with its action could theoretically allow hA3G to escape degradation and thus restrict virus proliferation [2],[19],[21]. However, an increased rate of G-to-A mutations compared to that from the error prone RT enzyme, as a result of an incomplete block of Vif, could potentially be used by HIV-1 to accelerate evolution of drug resistance and immune escape [30]. Variation at G-nucleotide sites is not uncommon within a HIV-1 population and polymorphism at HIV-1 drug resistance sites has been observed in single genome sequencing studies [31],[32]. Here, we used our model to estimate the chance for HIV-1 to benefit from such increased genetic variation due to hA3G-introduced mutations. G-to-A-induced drug resistance has been described, but until now its discussion has focused on GG-dinucleotide contexts [30],[33]. Our *in silico* model instead uses the extended information of the sequence context surrounding the mutation site and enabled us to calculate a probability score for each possible site. This approach enabled us to screen the entire HIV-1 genome and estimate the likelihood of mutations in the context of the dual gradients arising during reverse transcription [9],[10]. The moderate number of hA3G-mediated G-to-A mutations at known drug resistance sites implies that the hA3G-Vif interaction can be a potential target for new antiviral drugs, with little or no concern for enhancement or appearance of resistance to other antiviral drugs. The increased rate of G-to-A mutations from hA3G does not seem overly problematic for the evolution of drug resistance in HIV-1. However, as pointed out by Mulder et al, [34] it cannot be entirely excluded that other A3-induced changes in high scoring hA3G target sites in the vicinity of current drug targets (Figure S2) not associated with drug resistance to date, might give rise to compensatory or even new resistance mutations. In that study, the authors describe a mutation, M184I in RT, which emerged in a partially impaired HIV-1 vif mutant. The conclusion that hA3G was causing this mutation was based on the dinucleotide context and the results are somewhat contradictory to our findings (Figure 5). Given that G-to-A mutations are the most common RT errors and our previous experience and support from larger mutation site contexts derived from the literature, we emphasize that such a short dinucleotide context is unlikely to be sufficient to readily distinguish *bona fide* hA3G induced G-to-A mutations from RT error.

In conclusion, we have found evidence that low levels of APOBEC3G-induced mutagenesis have affected HIV-1 evolution, leading to enhanced rates of variation at sites with a high probability of match to the optimum context for hA3G-mediated deamination. The low levels of G-to-A mutation could allow the viruses to achieve enhanced genetic variation. However, since the predicted effect on resistance to standard antiviral drugs is likely to be small, we propose that concerns over increased resistance mutations should not impede development of HIV-1 Vif as a candidate drug target.

## Supporting Information

Figure S1 Position-specific scoring matrix (PSSM) derived from observed G-to-A mutation site contexts found in the literature.
(0.06 MB PDF)

Figure S2 HIV-1 (AF033819) *gag*, *pol* and *env* polyprotein ORFs and amino acid translations. Lower panel black bars indicate calculated hA3G context probabilities for each. Upper panels show stacked bars for accumulated number of simulated mutations. Red bars indicate nonsynonymous (i.e. change in amino acid) mutations and blue bars indicate synonymous mutations. Dark shaded bars (red and blue) represent simulations using an 80% probability cutoff for mutations and lighter shaded bars (orange and light blue) represent simulations using all context probabilities. Known resistance mutations are shown as shaded codons and amino acids. Grey shading represents amino acid changes due to mutations other than G-to-A editing which are shaded in red.
(0.30 MB PDF)

## References

[1] Esnault C, Heidmann O, Delebecque F, Dewannieux M, Ribet D (2005). APOBEC3G cytidine deaminase inhibits retrotransposition of endogenous retroviruses.. Nature.

[2] Harris RS, Liddament MT (2004). Retroviral restriction by APOBEC proteins.. Nat Rev Immunol.

[3] Okeoma CM, Lovsin N, Peterlin BM, Ross SR (2007). APOBEC3 inhibits mouse mammary tumour virus replication in vivo.. Nature.

[4] Telesnitsky A, Goff SP, Coffin JM, Hughes SH, Varmus HE (1997). Reverse Transcriptase and the Generation of Retroviral DNA.. Retroviruses.

[5] Bishop KN, Holmes RK, Sheehy AM, Davidson NO, Cho SJ (2004). Cytidine deamination of retroviral DNA by diverse APOBEC proteins.. Curr Biol.

[6] Harris RS, Bishop KN, Sheehy AM, Craig HM, Petersen-Mahrt SK (2003). DNA deamination mediates innate immunity to retroviral infection.. Cell.

[7] Mangeat B, Turelli P, Caron G, Friedli M, Perrin L (2003). Broad antiretroviral defence by human APOBEC3G through lethal editing of nascent reverse transcripts.. Nature.

[8] Zhang H, Yang B, Pomerantz RJ, Zhang C, Arunachalam SC (2003). The cytidine deaminase CEM15 induces hypermutation in newly synthesized HIV-1 DNA.. Nature.

[9] Yu Q, Konig R, Pillai S, Chiles K, Kearney M (2004). Single-strand specificity of APOBEC3G accounts for minus-strand deamination of the HIV genome.. Nat Struct Mol Biol.

[10] Suspene R, Rusniok C, Vartanian JP, Wain-Hobson S (2006). Twin gradients in APOBEC3 edited HIV-1 DNA reflect the dynamics of lentiviral replication.. Nucleic Acids Res.

[11] Jern P, Stoye JP, Coffin JM (2007). Role of APOBEC3 in genetic diversity among endogenous murine leukemia viruses.. PLoS Genet.

[12] Chiu YL, Greene WC (2008). The APOBEC3 Cytidine Deaminases: An Innate Defensive Network Opposing Exogenous Retroviruses and Endogenous Retroelements.. Annu Rev Immunol.

[13] Rulli SJ, Mirro J, Hill SA, Lloyd P, Gorelick RJ (2008). Interactions of Murine Apobec3 and Human Apobec3g with Murine Leukemia Viruses.. J Virol.

[14] Suspene R, Sommer P, Henry M, Ferris S, Guetard D (2004). APOBEC3G is a single-stranded DNA cytidine deaminase and functions independently of HIV reverse transcriptase.. Nucleic Acids Res.

[15] Henikoff JG, Henikoff S (1996). Using substitution probabilities to improve position-specific scoring matrices.. Comput Appl Biosci.

[16] Johnson VA, Brun-Vezinet F, Clotet B, Gunthard HF, Kuritzkes DR (2007). Update of the drug resistance mutations in HIV-1: 2007.. Top HIV Med.

[17] Russell RA, Moore MD, Hu WS, Pathak VK (2009). APOBEC3G induces a hypermutation gradient: purifying selection at multiple steps during HIV-1 replication results in levels of G-to-A mutations that are high in DNA, intermediate in cellular viral RNA, and low in virion RNA.. Retrovirology.

[18] Russell RA, Pathak VK (2007). Identification of two distinct human immunodeficiency virus type 1 Vif determinants critical for interactions with human APOBEC3G and APOBEC3F.. J Virol.

[19] Harris RS (2008). Enhancing immunity to HIV through APOBEC.. Nat Biotechnol.

[20] Mehle A, Wilson H, Zhang C, Brazier AJ, McPike M (2007). Identification of an APOBEC3G binding site in human immunodeficiency virus type 1 Vif and inhibitors of Vif-APOBEC3G binding.. J Virol.

[21] Nathans R, Cao H, Sharova N, Ali A, Sharkey M (2008). Small-molecule inhibition of HIV-1 Vif.. Nat Biotechnol.

[22] Holmes RK, Malim MH, Bishop KN (2007). APOBEC-mediated viral restriction: not simply editing?. Trends Biochem Sci.

[23] Jern P, Coffin JM (2008). Host-retrovirus arms race: trimming the budget.. Cell Host Microbe.

[24] OhAinle M, Kerns JA, Li MM, Malik HS, Emerman M (2008). Antiretroelement activity of APOBEC3H was lost twice in recent human evolution.. Cell Host Microbe.

[25] Berkhout B, de Ronde A (2004). APOBEC3G versus reverse transcriptase in the generation of HIV-1 drug-resistance mutations.. Aids.

[26] Muller V, Bonhoeffer S (2005). Guanine-adenine bias: a general property of retroid viruses that is unrelated to host-induced hypermutation.. Trends Genet.

[27] Keele BF, Giorgi EE, Salazar-Gonzalez JF, Decker JM, Pham KT (2008). Identification and characterization of transmitted and early founder virus envelopes in primary HIV-1 infection.. Proc Natl Acad Sci U S A.

[28] Armitage AE, Katzourakis A, de Oliveira T, Welch JJ, Belshaw R (2008). Conserved footprints of APOBEC3G on Hypermutated human immunodeficiency virus type 1 and human endogenous retrovirus HERV-K(HML2) sequences.. J Virol.

[29] Lee YN, Malim MH, Bieniasz PD (2008). Hypermutation of an ancient human retrovirus by APOBEC3G.. J Virol.

[30] Pillai SK, Wong JK, Barbour JD (2008). Turning up the volume on mutational pressure: is more of a good thing always better? (A case study of HIV-1 Vif and APOBEC3).. Retrovirology.

[31] Kearney M, Palmer S, Maldarelli F, Shao W, Polis MA (2008). Frequent polymorphism at drug resistance sites in HIV-1 protease and reverse transcriptase.. Aids.

[32] Palmer S, Kearney M, Maldarelli F, Halvas EK, Bixby CJ (2005). Multiple, linked human immunodeficiency virus type 1 drug resistance mutations in treatment-experienced patients are missed by standard genotype analysis.. J Clin Microbiol.

[33] Hache G, Mansky LM, Harris RS (2006). Human APOBEC3 proteins, retrovirus restriction, and HIV drug resistance.. AIDS Rev.

[34] Mulder LC, Harari A, Simon V (2008). Cytidine deamination induced HIV-1 drug resistance.. Proc Natl Acad Sci U S A.

