# Supplementary material for: Likely Role of APOBEC3G-Mediated G-to-A Mutations in HIV-1 Evolution and Drug Resistance
Source: PLoS Pathog. 2009 Apr 3;5(4):e1000367. doi: 10.1371/journal.ppat.1000367 (PMC2659435; doi:10.1371/journal.ppat.1000367)
Supplement: Figure S1 — Position-specific scoring matrix (PSSM) derived from observed G-to-A mutation site contexts found in the literature. (0.06 MB PDF) [file ppat.1000367.s001.pdf]

FIGURE S1

Summarized observed mutation contexts

G-to-A mutation contexts derived from refs [1, 2].

Observed	Pos	-3	-2	-1	0	1	2	3	4	5
	A	246	190	268	0	119	598	620	825	584
	C	124	239	114	0	16	49	12	0	0
	G	350	310	655	1324	1161	635	370	59	149
	T	294	275	287	0	28	42	12	86	237
	**sum**	**1014**	**1014**	**1324**	**1324**	**1324**	**1324**	**1014**	**970**	**970**

Expected frequencies (%)	
A	37.8
C	17.2
G	16.3
T	28.7

χ^2 test (1 df)

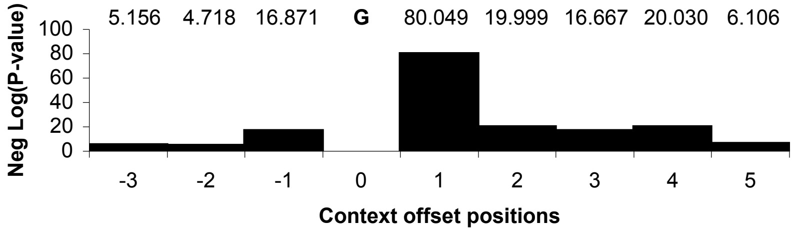

Context PSSM (compensated for sampling errors)

Position-specific scoring matrix was derived using equations 5, 6, and 7 in ref [3].

LOG(((Nc / (Nc + sqrt(N)) * (nc / Nc)) + ((sqrt(N)) / (Nc + sqrt(N))) * (((sqrt(N)) * prob(nt)) / sqrt(N))) / prob(nt))

N	Total number observed nucleotides at PSSM position
Nc	Observed number of specific nucleotides (A, C, G or T) at PSSM position
nc	Expected nucleotide frequency (A, C, G or T)

PSSM position	-3	-2	-1	0	1	2	3	4	5
A	-0.185	-0.292	-0.261		-0.588	0.075	0.204	0.345	0.197
C	-0.143	0.133	-0.289		-1.022	-0.627	-1.013	-1.507	-1.507
G	0.319	0.267	0.474	0.778	0.721	0.461	0.343	-0.406	-0.025
T	0.004	-0.024	-0.118		-1.007	-0.872	-1.152	-0.481	-0.068
**max**	0.319	0.267	0.474	0.778	0.721	0.461	0.343	0.345	0.197
**max sum**	**3.904**								
**min**	-0.185	-0.292	-0.289	0.778	-1.022	-0.872	-1.152	-1.507	-1.507
**min sum**	**-6.048**								

References

1 Suspene R, Sommer P, Henry M, Ferris S, Guetard D, et al. (2004) APOBEC3G is a single-stranded DNA cytidine deaminase and functions independently of HIV reverse transcriptase. Nucleic Acids Res 32: 2421-2429.

2 Yu Q, Konig R, Pillai S, Chiles K, Kearney M, et al. (2004) Single-strand specificity of APOBEC3G accounts for minus-strand deamination of the HIV genome. Nat Struct Mol Biol 11: 435-442.

3 Henikoff JG, Henikoff S (1996) Using substitution probabilities to improve position-specific scoring matrices. Comput Appl Biosci 12: 135-143.
