# Supplementary figures and images for: Likely Role of APOBEC3G-Mediated G-to-A Mutations in HIV-1 Evolution and Drug Resistance
Source: PLoS Pathog. 2009 Apr 3;5(4):e1000367. doi: 10.1371/journal.ppat.1000367 (PMC2659435; doi:10.1371/journal.ppat.1000367)

HIV-1 (AF033819)

*gag*

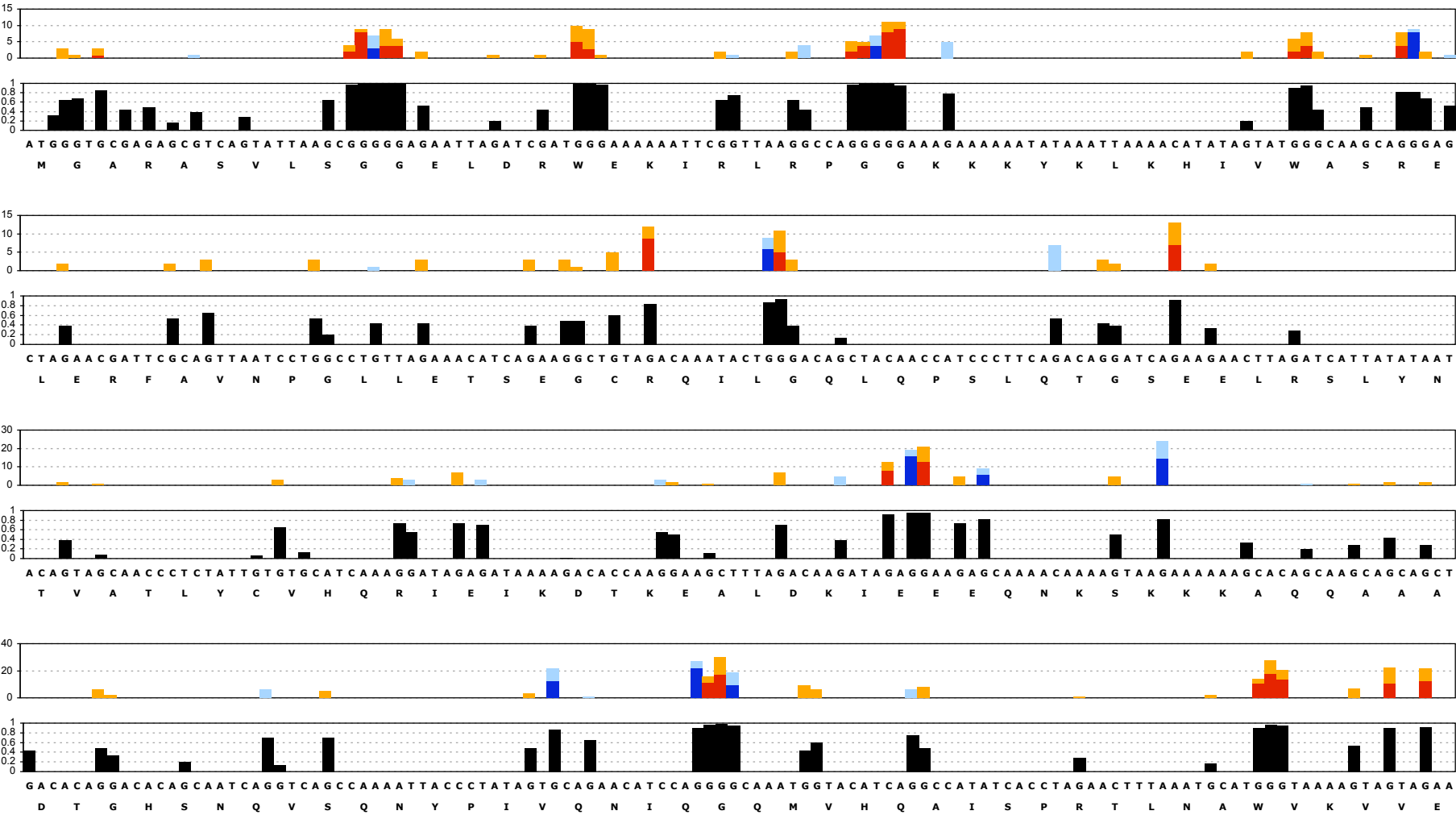

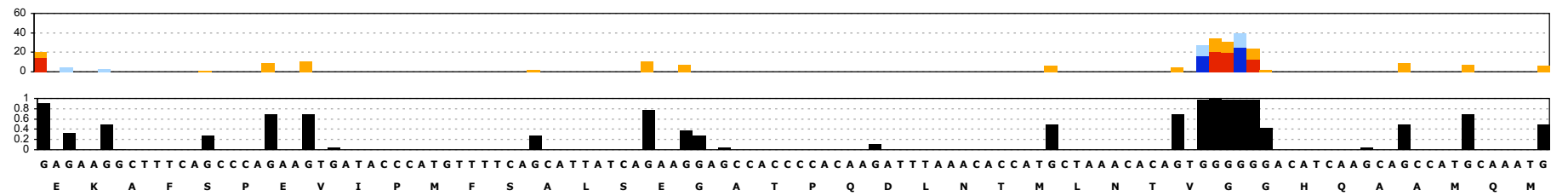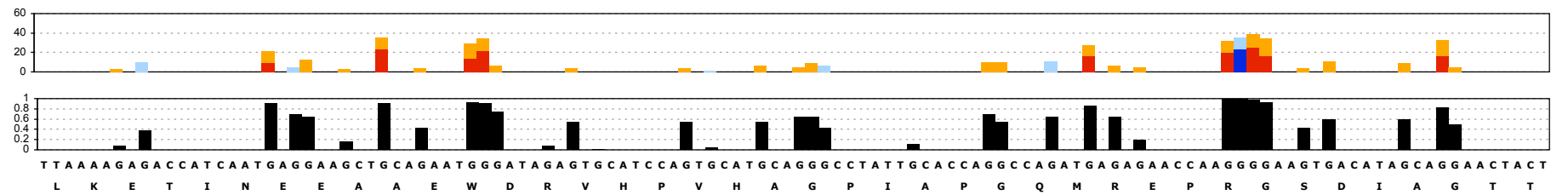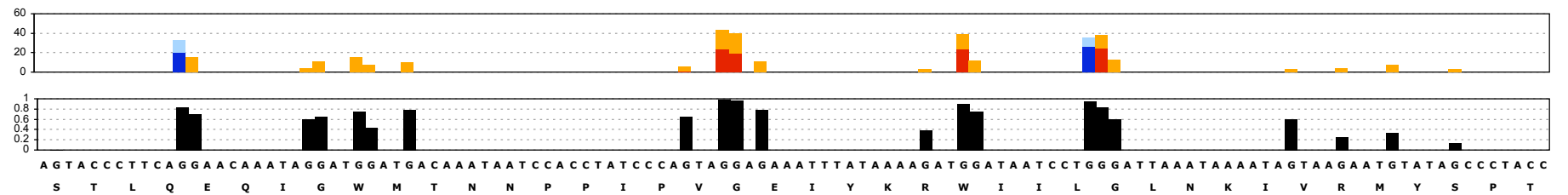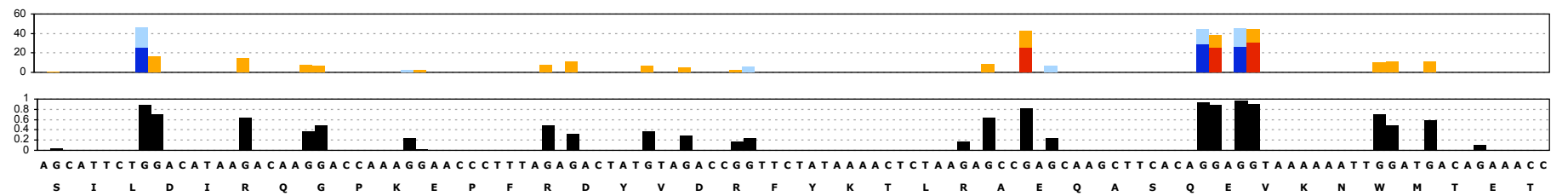

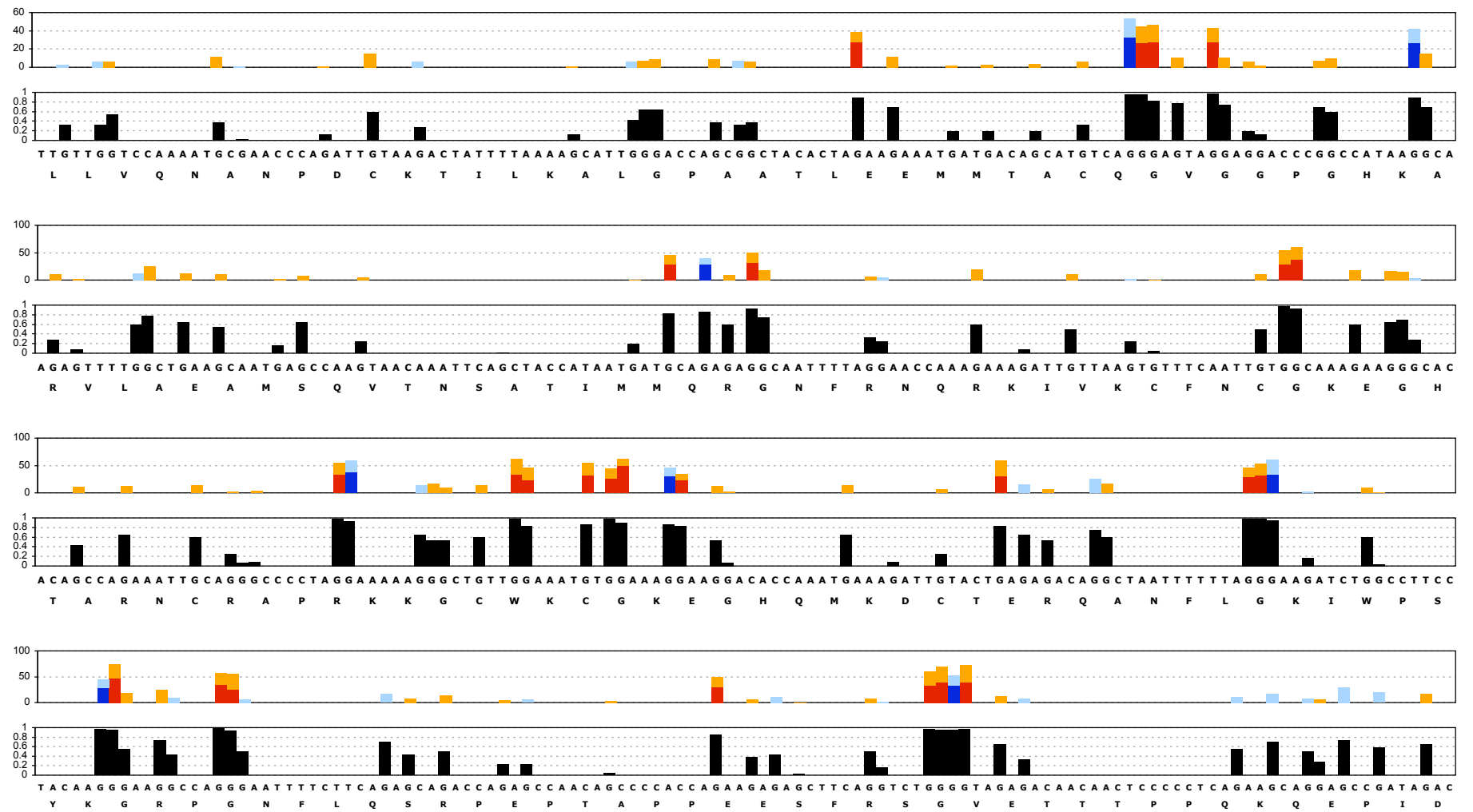

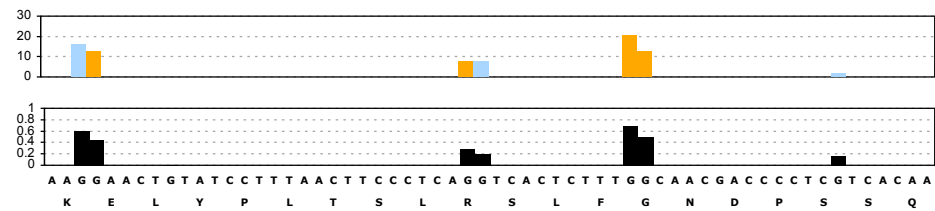

HIV-1 (AF033819)

*pol*

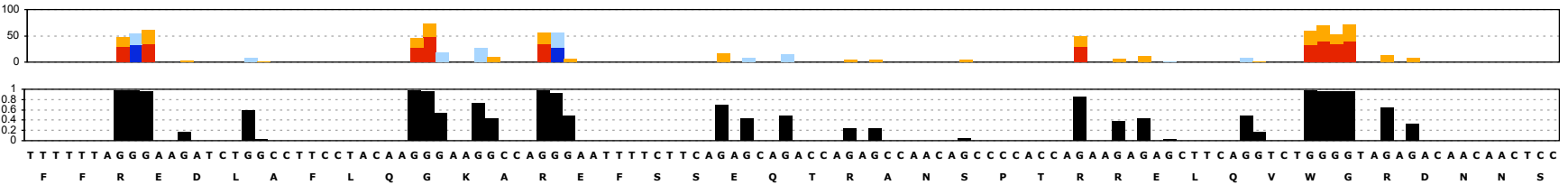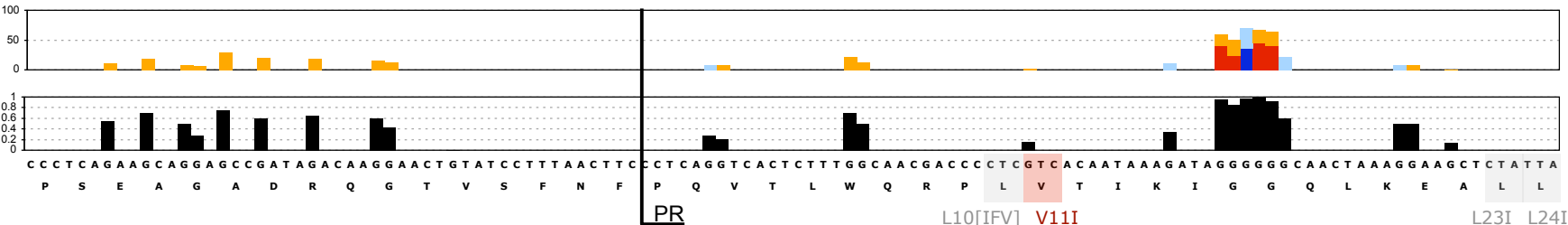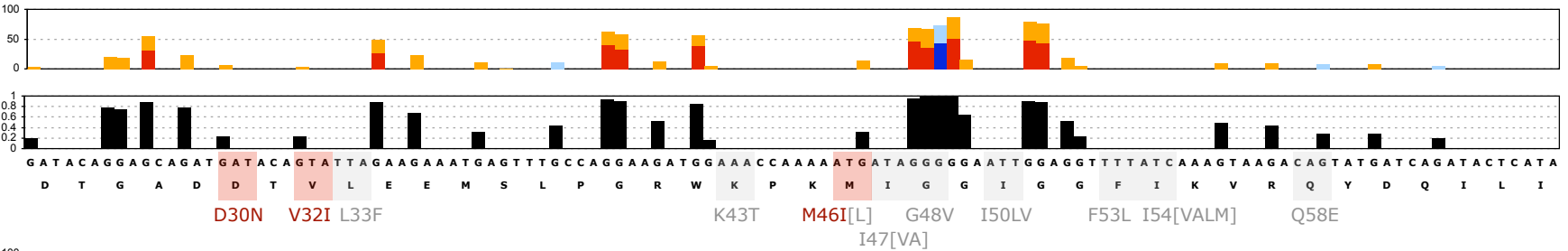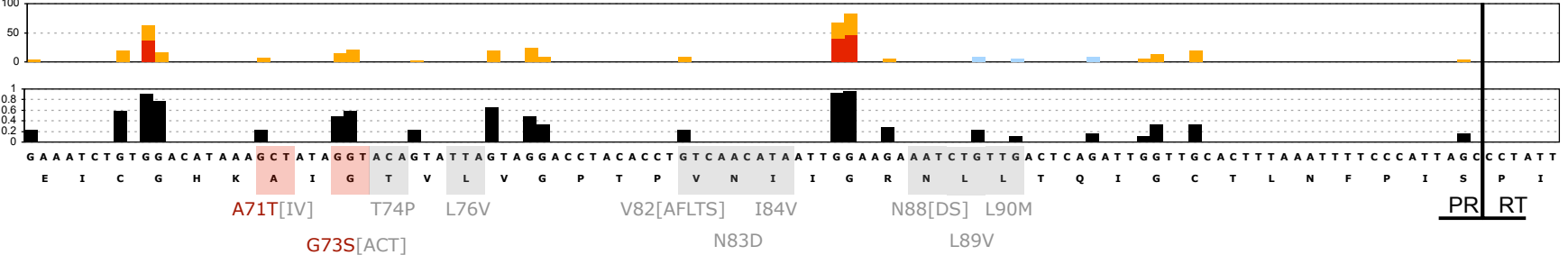

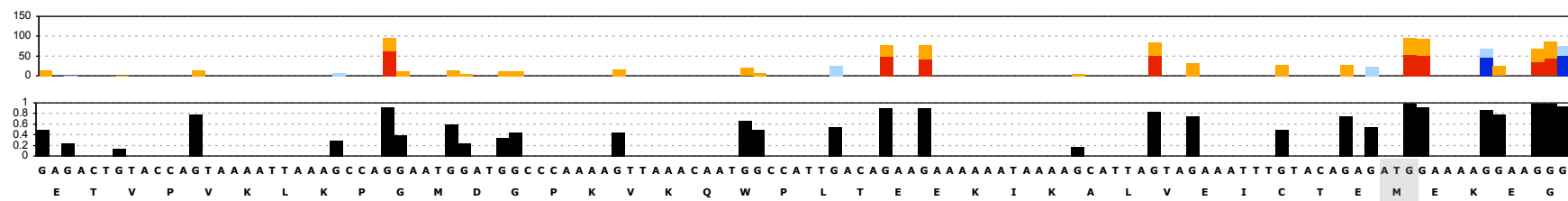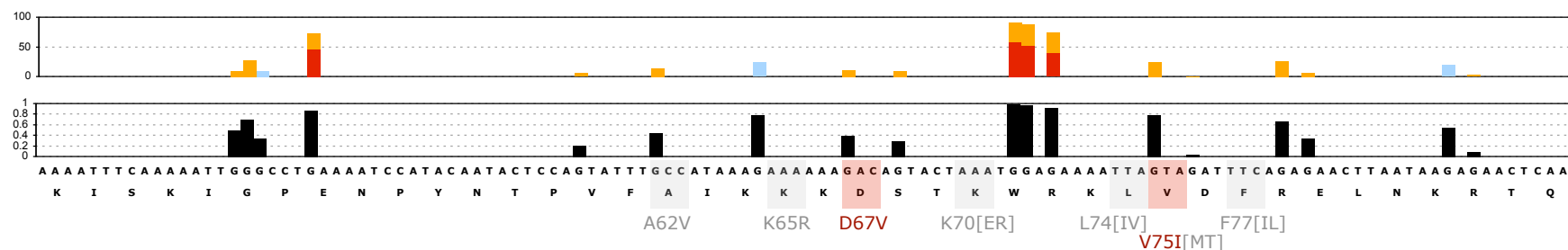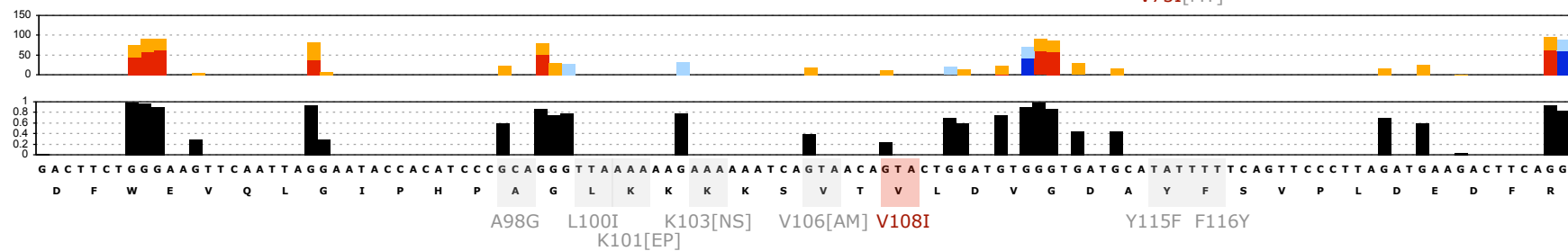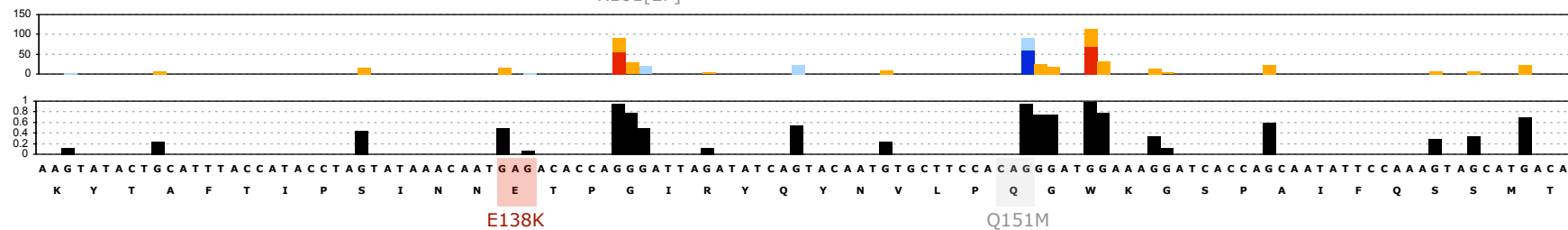

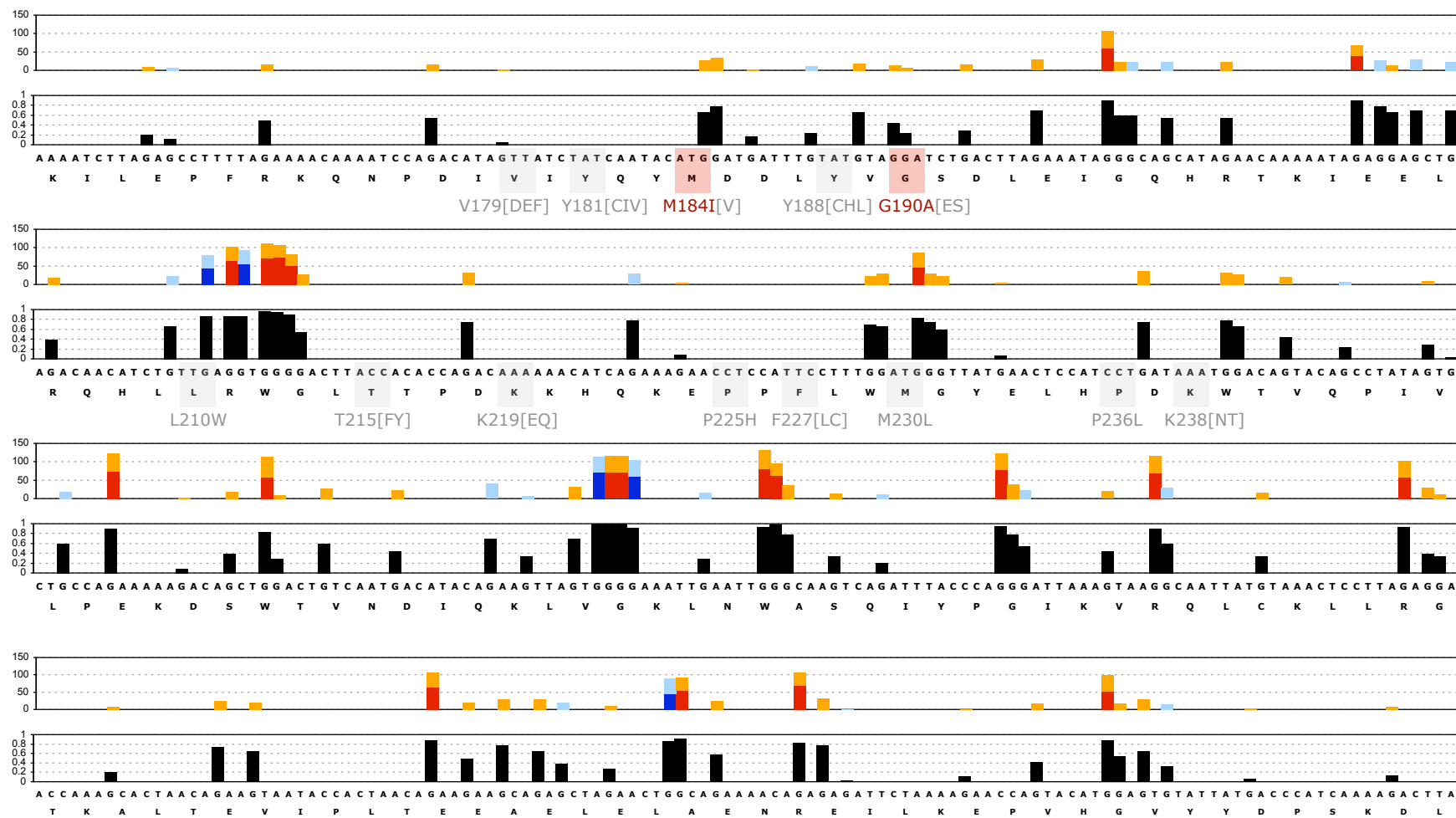

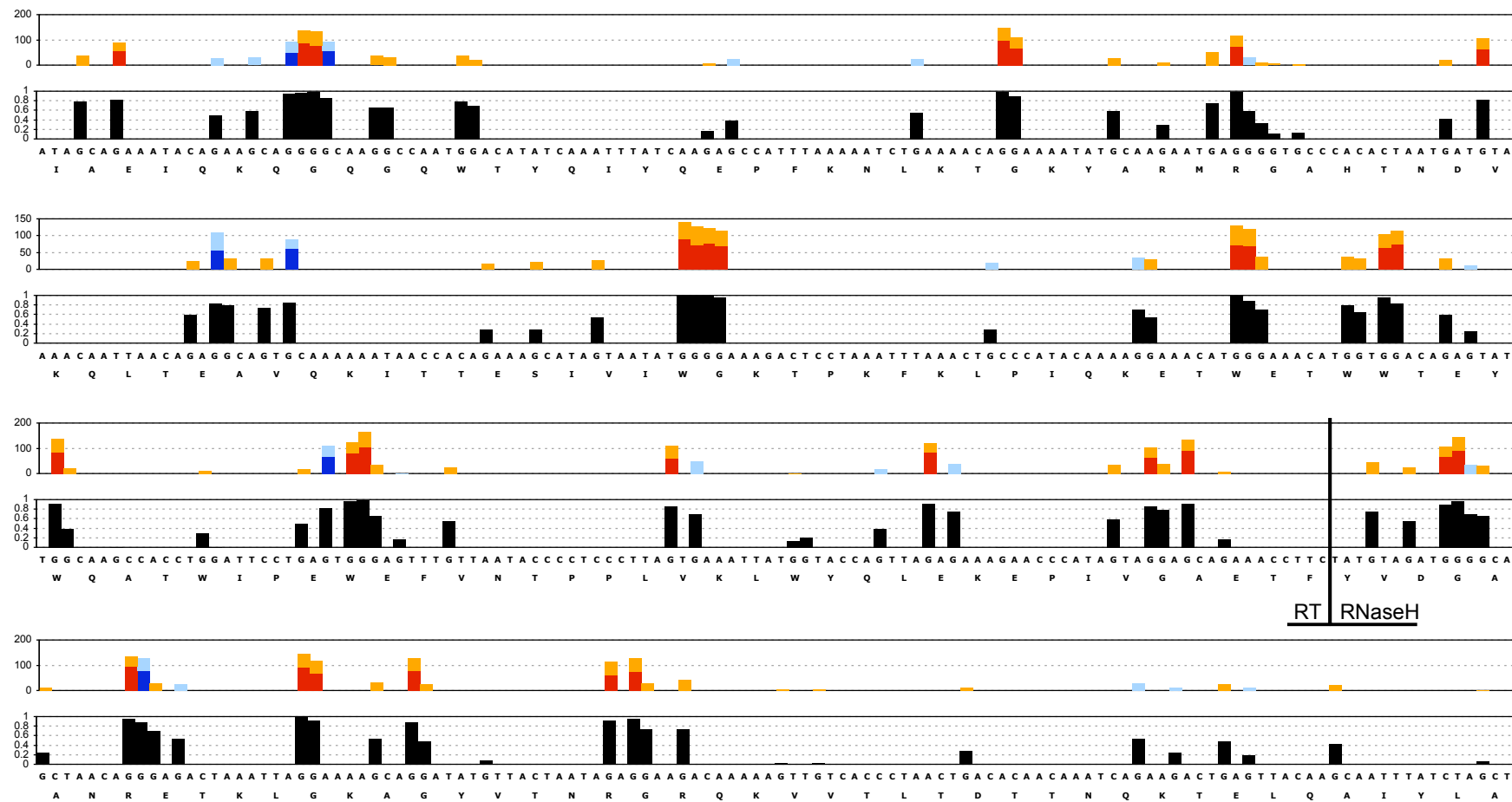

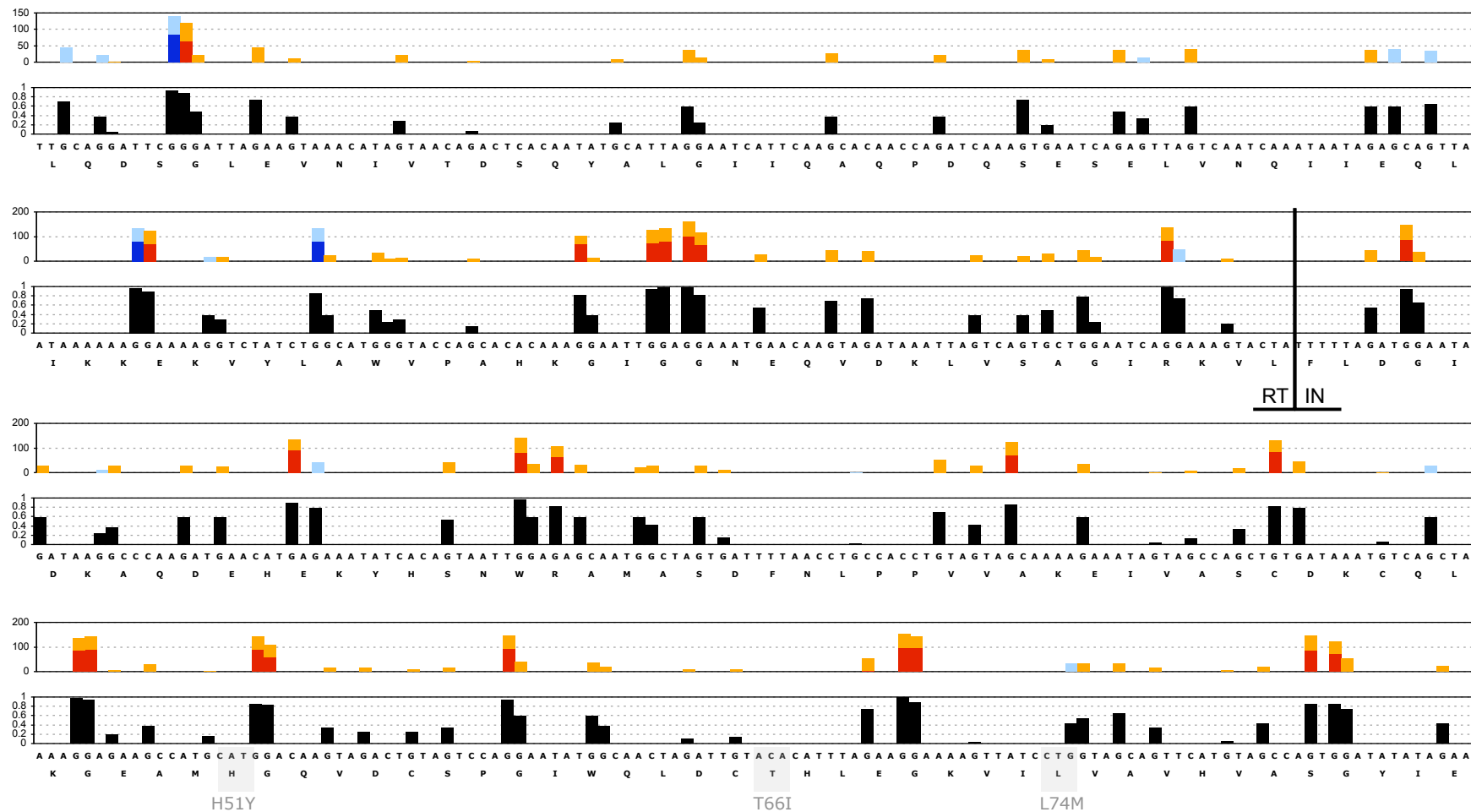

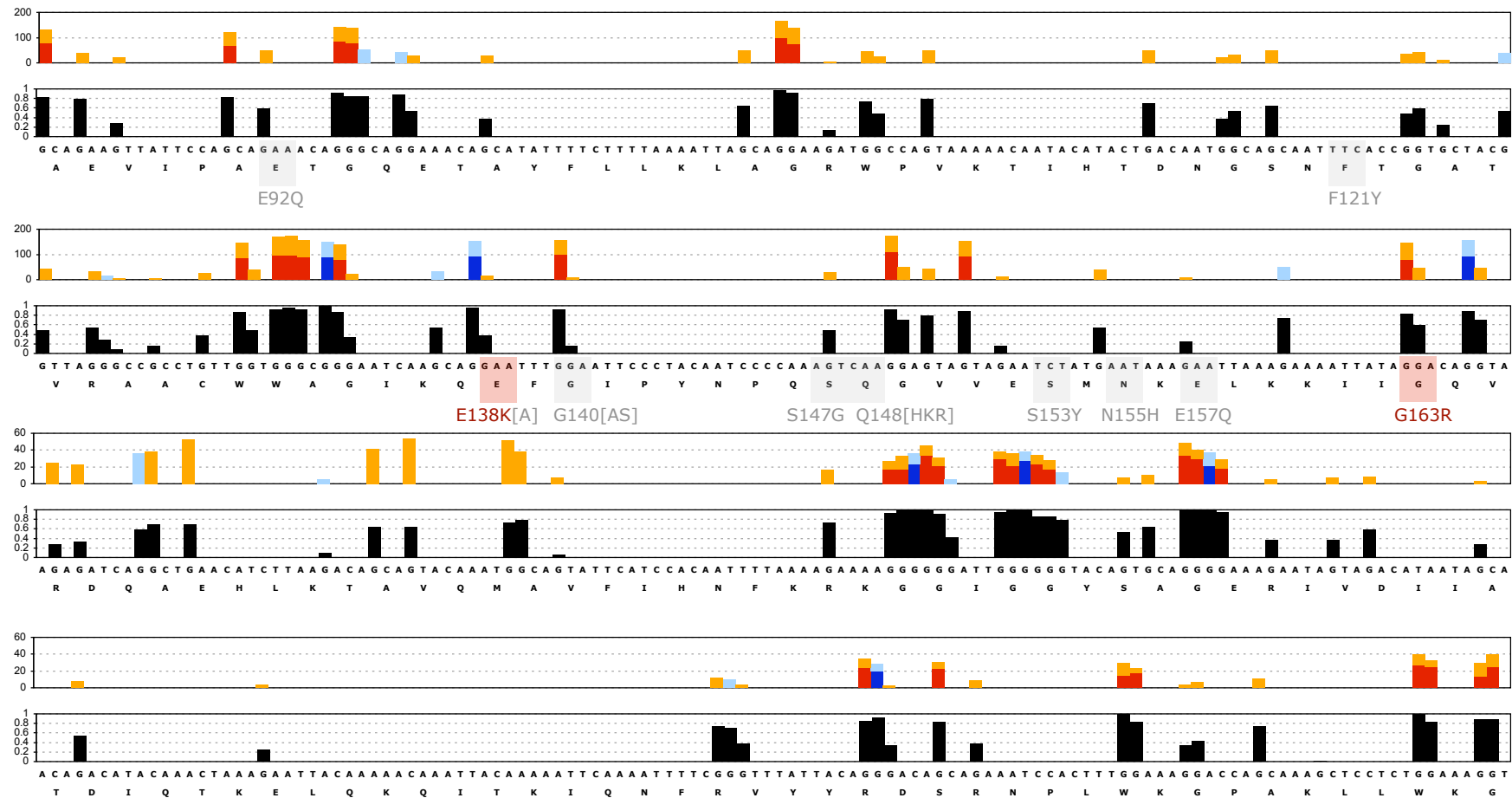

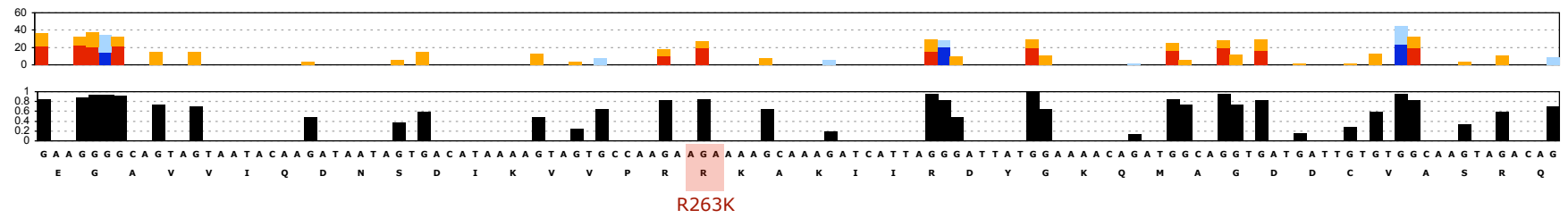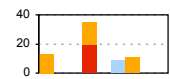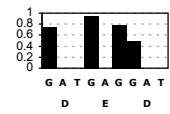

HIV-1 (AF033819)

env

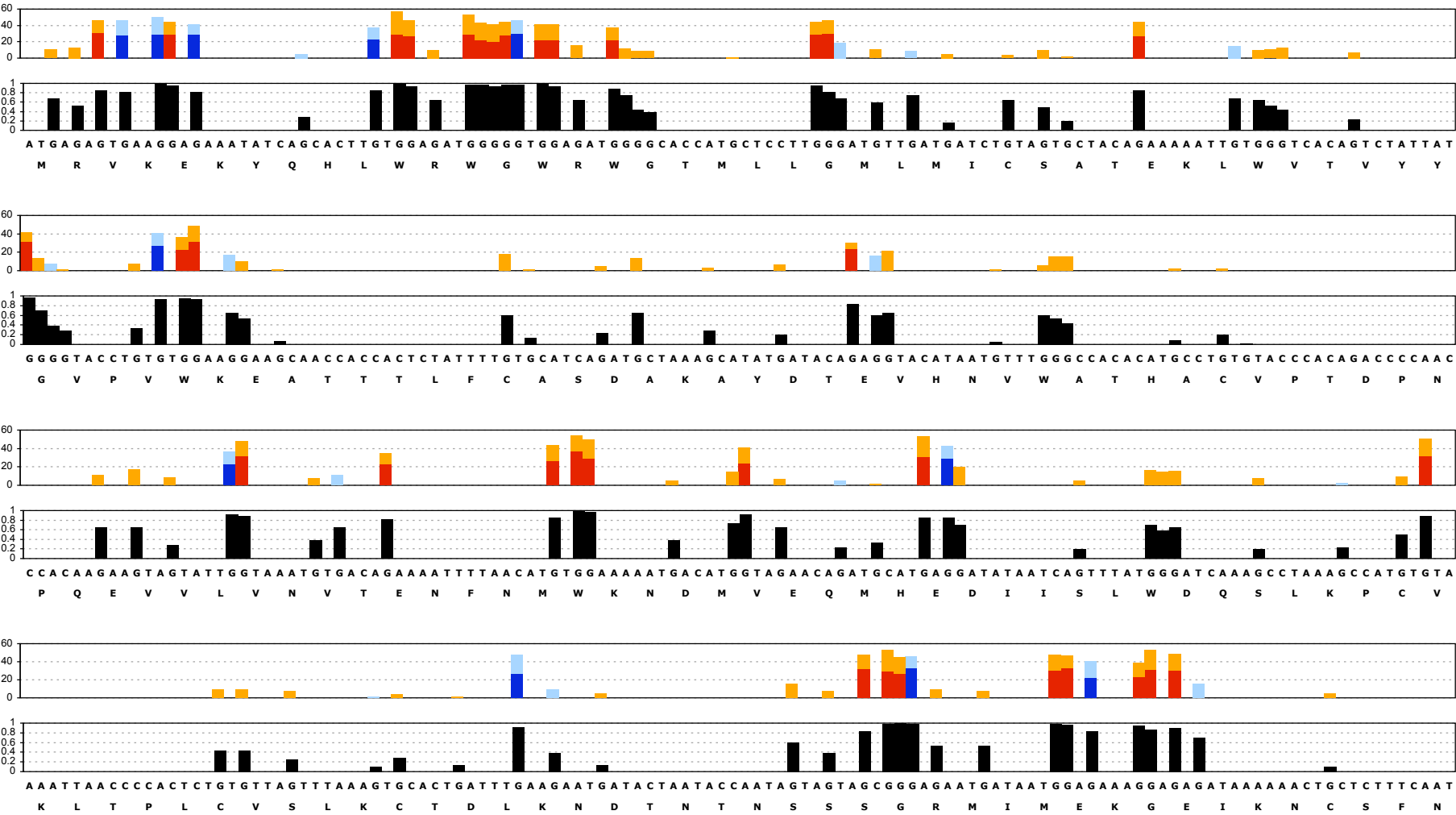

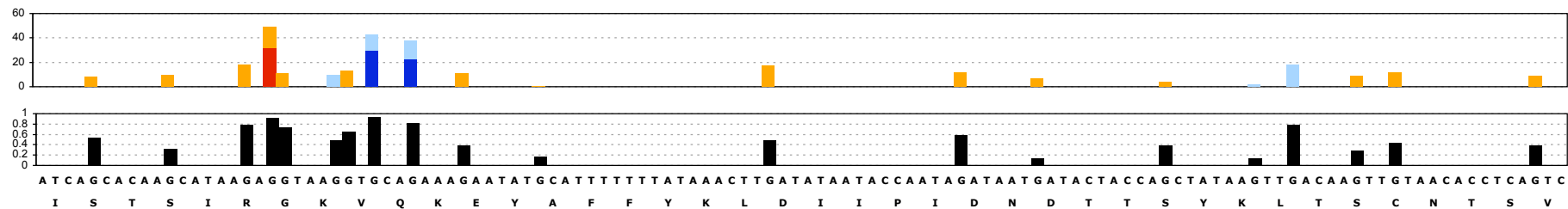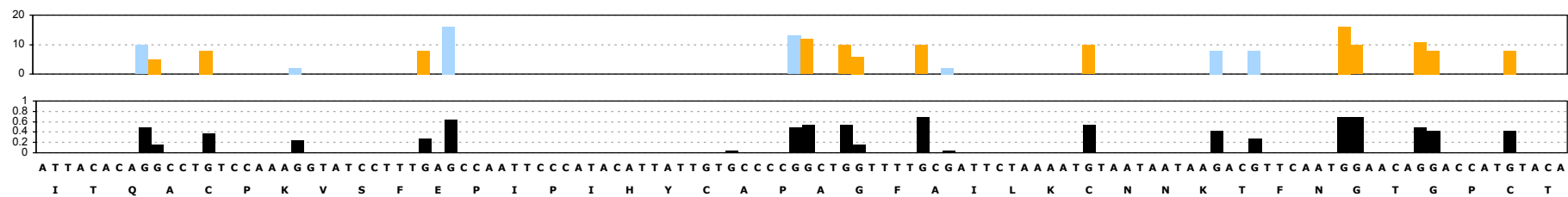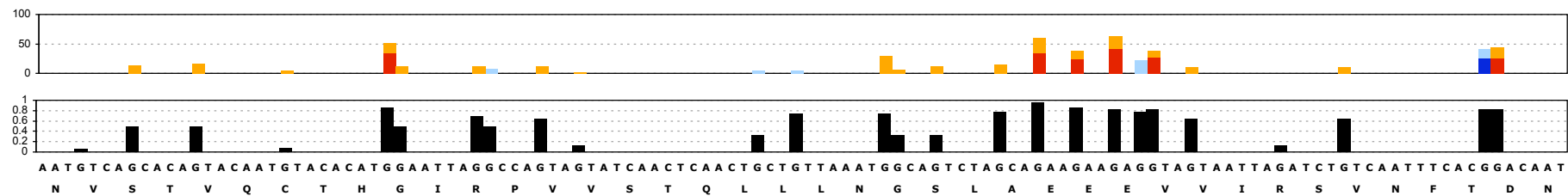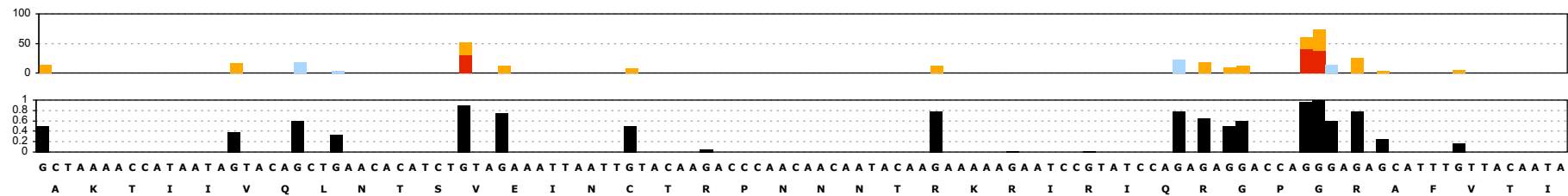

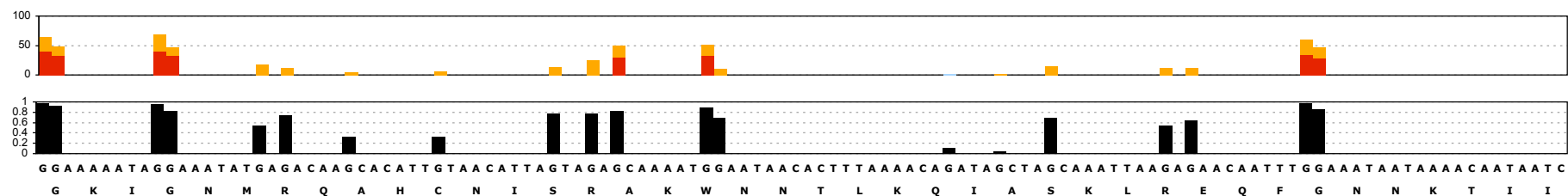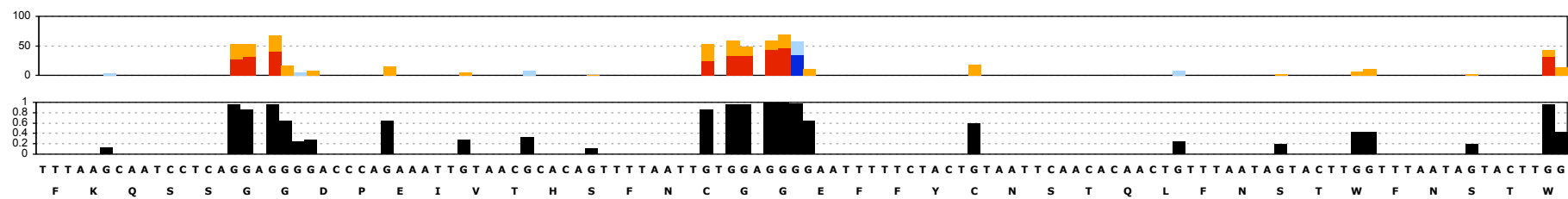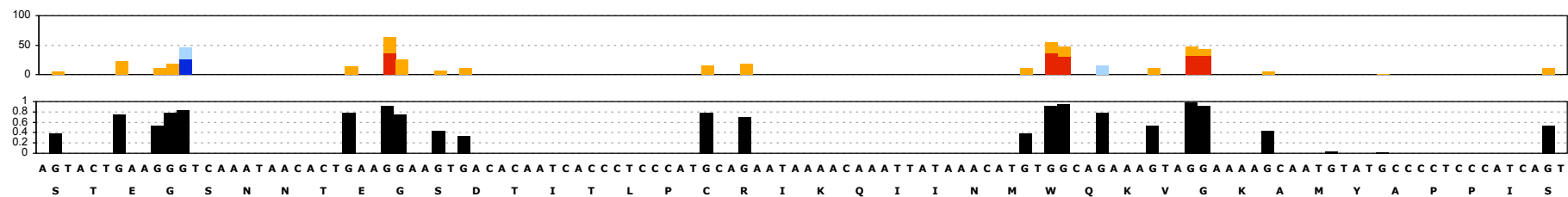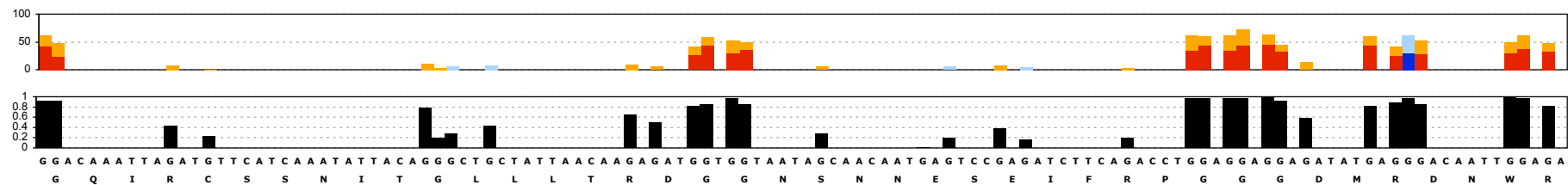

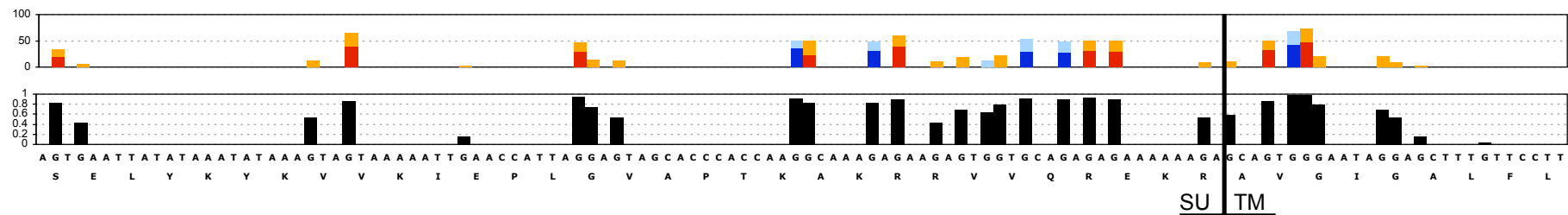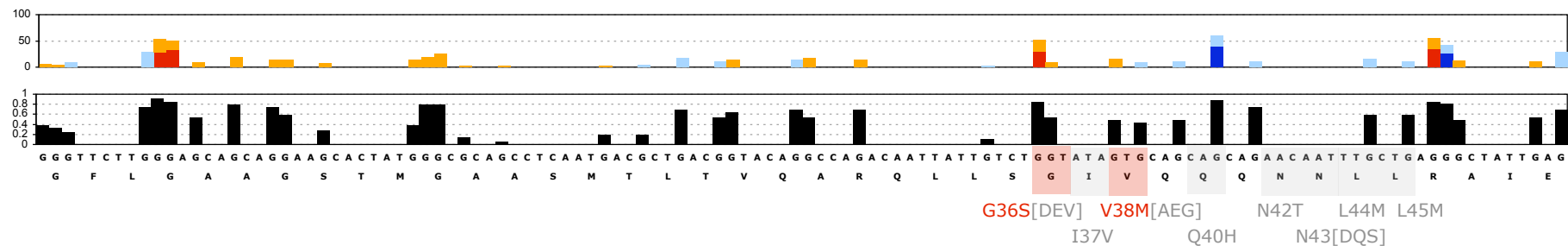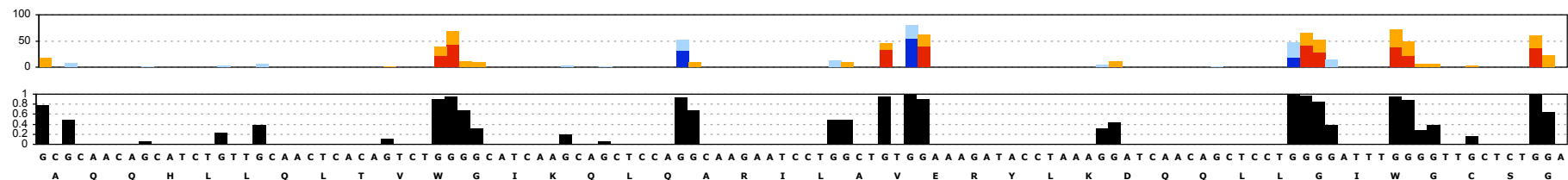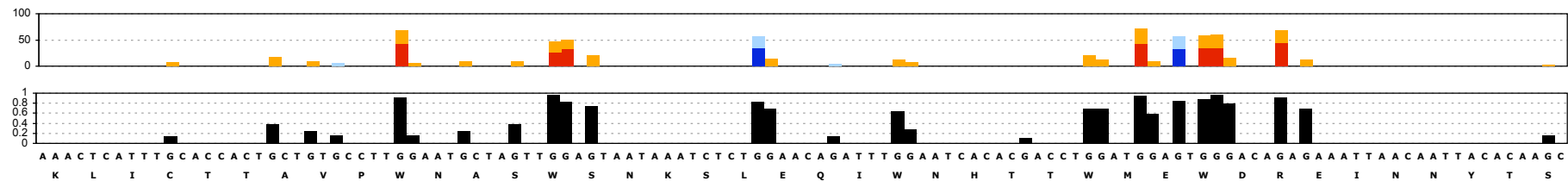

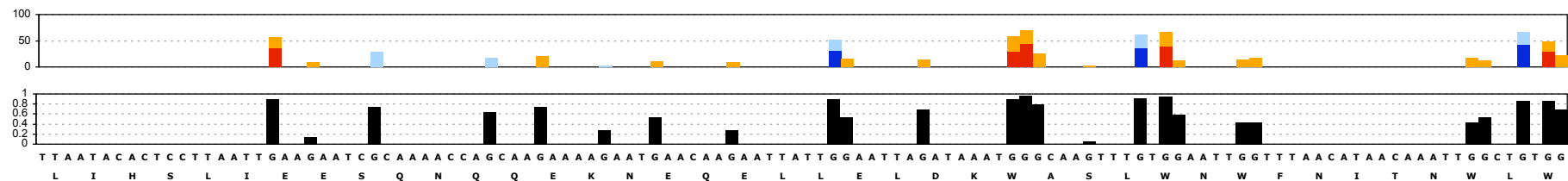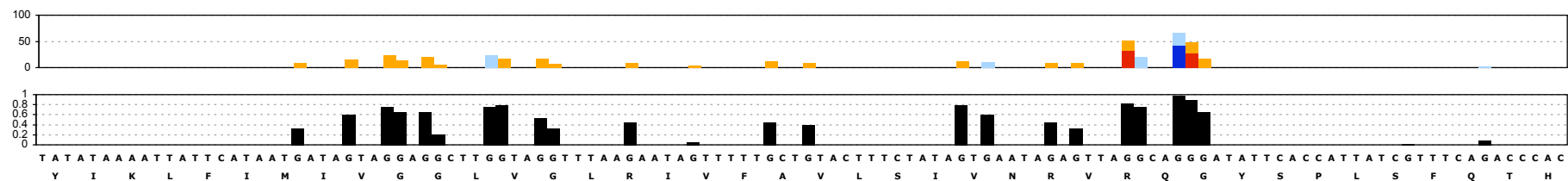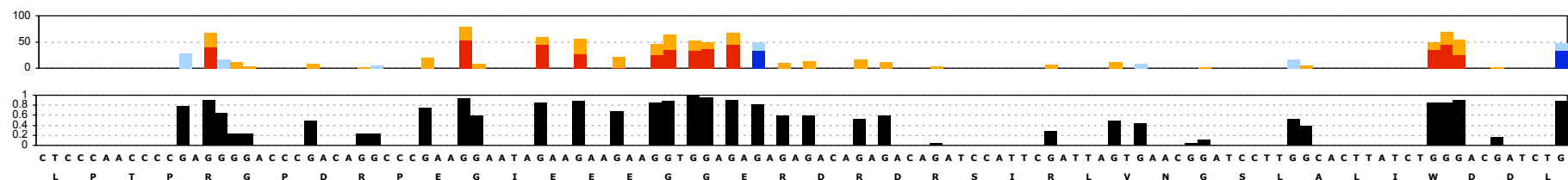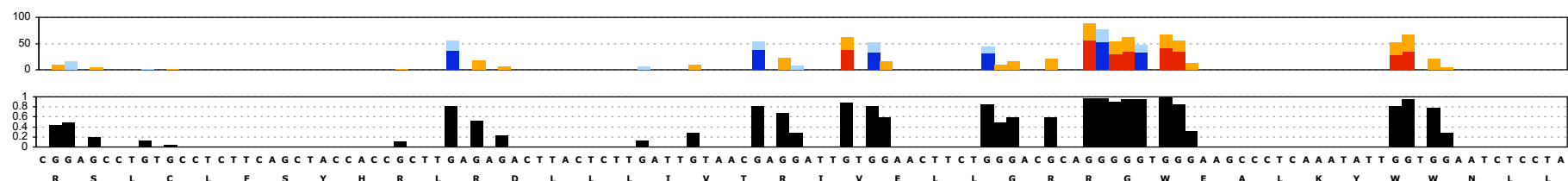

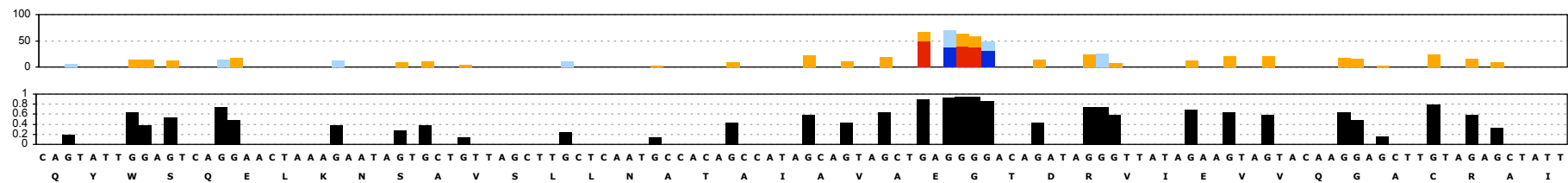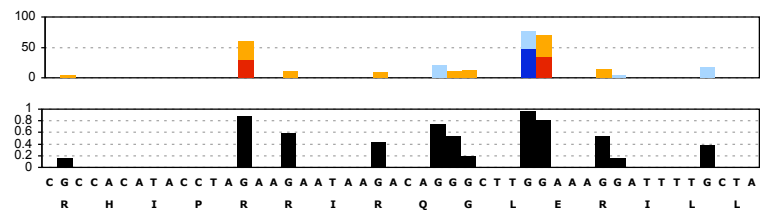

Supplement: Figure S2 — HIV-1 (AF033819) gag, pol and env polyprotein ORFs and amino acid translations. Lower panel black bars indicate calculated hA3G context probabilities for each. Upper panels show stacked bars for accumulated number of simulated mutations. Red bars indicate nonsynonymous (i.e. change in amino acid) mutations and blue bars indicate synonymous mutations. Dark shaded bars (red and blue) represent simulations using an 80% probability cutoff for mutations and lighter shaded bars (orange and light blue) represent simulations using all context probabilities. Known resistance mutations are shown as shaded codons and amino acids. Grey shading represents amino acid changes due to mutations other than G-to-A editing which are shaded in red. (0.30 MB PDF) [file ppat.1000367.s002.pdf]
